# Genome editing approaches with CRISPR/Cas9: the association of NOX4 expression in breast cancer patients and effectiveness evaluation of different strategies of CRISPR/Cas9 to knockout Nox4 in cancer cells

**DOI:** 10.1186/s12885-023-11183-9

**Published:** 2023-11-27

**Authors:** Marzieh Javadi, Hossein Sazegar, Abbas Doosti

**Affiliations:** 1 Department of Biology, Faculty of Science, Shahrekord Branch, Islamic Azad University, Shahrekord, Iran; 2 Biotechnology Research Center, Shahrekord Branch, Islamic Azad University, Shahrekord, Iran

**Keywords:** Breast cancer, NOX4, CRISPR/Cas9, Knockout

## Abstract

**Background:**

The increasing prevalence of cancer detection necessitated practical strategies to deliver highly accurate, beneficial, and dependable processed information together with experimental results. We deleted the cancer biomarker NOX4 using three novel genetic knockout (KO) methods. Homology-directed repair (*HDR*), Dual allele HITI (Du-HITI) and CRISPR-excision were utilized in this study.

**Methods:**

The predictive value of the NOX4 expression profile was assessed using a combined hazard ratio (HR) with a 95% confidence interval (CI). With a 95% confidence interval, a pooled odd ratio (OR) was used to calculate the relationship between NOX4 expression patterns and cancer metastasis. There were 1060 tumor patients in all sixteen research that made up this meta-analysis. To stop the NOX4 from being transcribed, we employed three different CRISPR/Cas9-mediated knockdown methods. The expression of RNA was assessed using RT-PCR. We employed the CCK-8 assay, colony formation assays, and the invasion transwell test for our experiments measuring cell proliferation and invasion. Using a sphere-formation test, the stemness was determined. Luciferase reporter tests were carried out to verify molecular adhesion. Utilizing RT-qPCR, MTT, and a colony formation assay, the functional effects of NOX4 genetic mutation in CRISPR-excision, CRISPR-HDR, and CRISPR du-HITI knockdown cell lines of breast cancer were verified.

**Results:**

There were 1060 malignant tumors in the 16 studies that made up this meta-analysis. In the meta-analysis, higher NOX4 expression was linked to both a shorter overall survival rate (HR = 1.93, 95% CI 1.49–2.49, P < 0.001) and a higher percentage of lymph node metastases (OR = 3.22, 95% CI 2.18–4.29, P < 0.001). In breast carcinoma cells, it was discovered that NOX4 was overexpressed, and this increase was linked to a poor prognosis. The gain and loss-of-function assays showed enhanced NOX4 breast carcinoma cell proliferation, sphere-forming capacity, and tumor development. To activate transcription, the transcriptional factor E2F1 also attaches to the promoter region of the Nanog gene. The treatment group (NOX4 ablation) had substantially more significant levels of proapoptotic gene expression than the control group (P < 0.01). Additionally, compared to control cells, mutant cells expressed fewer antiapoptotic genes (P < 0.001). The du-HITI technique incorporated a reporter and a transcription termination marker into the two target alleles. Both donor vector preparation and cell selection were substantially simpler using this approach than with “CRISPR HDR” or “CRISPR excision.“ Furthermore, single-cell knockouts for both genotypes were created when this method was applied in the initial transfection experiment.

**Conclusions:**

The NOX4 Knockout cell lines generated in this research may be used for additional analytical studies to reveal the entire spectrum of NOX4 activities. The du-HITI method described in this study was easy to employ and could produce homozygous individuals who were knockout for a specific protein of interest.

**Supplementary Information:**

The online version contains supplementary material available at 10.1186/s12885-023-11183-9.

## Background

Breast cancer is one of the most common cancers in women worldwide and is the leading cause of female disease-related mortality [1, 2]. The most popular forms of breast cancer therapy were surgery, chemotherapeutic, and radiation therapy, considerably increasing therapeutic advantages [3]. Based on numerous research, breast cancer regeneration and survival have been connected to stem cell characteristics [4]. A subtype of malignant cells involved in the malignancy may be called cancer stem cells (CSCs) cells. CSCs might self-renew, differentiate in many directions, continue to divide indefinitely and heal malignancies. They were connected to radio-chemotherapy resistance, recurrence, cancer growth, multiplication, invasion, and metastasis [5]. Breast cancer stem cells (BCSCs) have been shown to have a significant role in the ability of cancer cells to reproduce and self-renew [6].

Reactive oxygen species (ROS) are involved in various biological systems as cytoplasmic signalling agents [7]. NADPH-oxidases regulate reactive oxygen species (ROS) production [8]. NADPH oxidases play various physiological roles, including cellular division, renal function control, and microbial immunological response; however, tumors are more likely to express them excessively than normal [9]. According to current research, NOX4 is a produced NOX variant crucial for synthesizing H2O2 and is a member of the NOX class [10]. A few studies demonstrate that NOX4 promotes cell growth through cell cycle regulation and apoptosis suppression, even if the methods through which NOX4 influences the proliferation, invasion, and survival of cancer cells are still unclear [11, 12].

Genome editing methods are now possible because of developments in molecular biology, allowing us to alter genomes and investigate how genetic modifications affect organisms’ operations [13]. Treatment for cancers impacting the body’s healthy cells has been carried out in various ways. Molecular biology research must continue to create effective cancer and oncology treatment strategies [14]. The Clustered Regularly Interspaced (CRI) protein system has recently been linked to Short Palindromic Repeats (CRISPR), a powerful new therapeutic method with great precision and efficacy for treating cancer, have been adopted by the national cancer institute to lower the number of fatalities brought on by cancer [15]. Numerous studies were planned and conducted to investigate the function of NOX4. Three different study kinds existed. Two antisense oligonucleotides were used to degrade produced RNA: RNA interference (RNAi) and antisense genomic regions. The second category includes CRISPR-mediated inhibition (CRISPRi) and activation (CRISPRa), which interact with the regulatory regions of the gene to control its degree of expression [14]. Genes in the third group were deleted using adaptable nucleases like CRISPR/Cas9 (CRISPR-associated protein-9 nuclease), transcription activator-like effector nucleases (TALENs), and zinc-finger nucleases (ZFNs). Numerous parameters were recommended [14, 15] for the ideal combination of a laboratory experiment for a particular transcription.

The main topics of these criteria were the targeted gene’s location and relationship to other genes, the positioning of its regulatory regions [15, 16], and biological materials’ sub-cellular location. To investigate a NOX4’s function, partial transcript reduction caused by the method’s structure, the gene’s nuclear location, and possible off-target effects were at least two limitations [17, 18]. The CRISPRi and CRISPRa techniques may not be able to inhibit or promote gene transcription when the expression of the target gene is regulated in a complex manner [19, 20]. The CRISPR/Cas9 dual genotype homology-independent targeted integration method used a targeting segment to replace the genomic region between two Cas9 double-strand breaks (DSBs). The biological roles of NOX4 in breast cancer development and the underlying biochemical mechanisms remain a mystery. The oncogene NOX4 impacted the tumor’s poor prognosis. The histone modification profile on p57 was dictated by an interaction between the histone methyl-transferase EZH2 and NOX4 [21]. It also functioned as an oncogene in cancer and pancreatic ductal adenocarcinoma [21–23]. After screening and assessing the situation, we decided on the NOX4 as the target. In the current investigation, NOX4 was discovered to be a carcinogenic agent in the growth of breast cancer tumors. In the current investigation, a CRISPR/Cas9 version was employed. This method replaced the genomic region between two Cas9-induced double-strand breaks (DSBs) in each genotype with the targeted segment.

## Materials and methods

### A. Bioinformatics analyzes

#### Search strategy and study selection

From April to June 2021, we read relevant and related publications in Medline, EMBASE, Web of Science, and Wanfang. The search term “NOX4” have been used. The articles that fit specified criteria were all added at once: (1) Participants were split into two groups according to their NOX4 transcription quantities (2) Risk ratios with 95% confidence intervals (CIs) for the association among NOX4 transcription and mortality percentages (3) The papers were published in English. The following conditions were not recommended:

- Research studies that were letters, abstracts from conferences, meta-analyses, or review publications.
- Research papers that used The Cancer Genome Atlas (TCGA) databases to analyze the prognostic importance of NOX4 level of transcription.
- Research papers that used the same subjects.

### Data collection and quality assessment

Duplicate studies were excluded according to the method of Kwon et al. (2015) [24]. Two writers independently obtained data from eligible research. All of the authors discussed any disagreements and agreed. The following information was retrieved: first author, time of publication, geography, cancer type, therapeutic classification, statistical significance, detection method, quality controls, and cut-off parameters; HRs for OS with 95% confidence intervals; logistic regression type; and follow-up duration. The multivariate HRs were picked first when HRs were obtained using both univariate and multivariate assessments because they had fewer confounding factors. If not explicitly stated in the paper, HRs with 95% CIs would be produced from Kaplan-Meier survival graphs using the Engauge Digitizer tool. The high and low NOX4 expression cutoffs used for the survival analysis were 1.92. The qualitative aspects of the research were evaluated using the Newcastle-Ottawa scale (NOS) criteria, which employed a star grading scale from 0 to 9 [25].

### Validation using data that is readily available to the public

The Cancer Genome Atlas (TCGA) publication standards were used in this study. Gene Expression Monitoring Interactive Modeling evaluated the relationships between NOX4 transcriptional level and OS and DFS (GEPIA). The logistic regression was computed using the K-M approach and log-rank analysis, and the HRs and p values were shown in the K-M curve diagrams [26].

### Detection of expression levels and co-expression of apoptotic genes

To identify the genes expressed differently to examine the significance of gene abnormal expression in breast cancer, reads were mapped to the reference genome (human: GRCh38) using CLC Genomics Workbench Version 21 allowing two mismatches to be utilized to identify the genes expressed differently to examine the significance of gene abnormal expression in breast cancer. The information for invasive breast carcinoma, comprised 16,137 samples and downloaded the RNA expression counts, RNA expression counts were collected from The Cancer Genome Atlas (TCGA) from the cBioPortal for Cancer Genomics (cbioportal.org) can compare white our data. The HUGO Gene Nomenclature Committee’s database provided the gene names and symbols (genenames.org). All the genes involved in the apoptosis pathway were initially obtained from the QIAGEN website (www.qiagen.com). It was done in the manner that gene expression was assessed in breast cancer to examine the co-expression of apoptotic genes highly expressed in breast cancer. Plots of the apoptotic genes with high expression and the gene expression network were made.

### Identification of miRNAs and genes with variable expression

The differentially expressed genes were predicted using a bioinformatics technique to examine aberrant gene expression’s function in breast cancer. The HUGO Gene Nomenclature Committee’s database was used to get the gene names and symbols (genenames.org). The database for invasive breast carcinoma, comprising 1,105 samples, was collected from The Cancer Genome Atlas (TCGA) from the cBioPortal for Cancer Genomics (cbioportal.org). The retrieved genes can be input, and the data can be submitted by choosing the cancer study and genomic profiles. A similar method might be used to choose miRNAs. In the supplementary material, the exact method of selecting and identifying miRNAs was described.

### Analysis of gene-miRNA interactions

The binding of genes to miRNAs was predicted utilizing bioinformatics software RegRNA 2.0 (regrna2.mbc.nctu.edu.tw/detection.html) to determine the miRNAs that could target genes. The National Center for Biotechnology Information’s GenBank database (ncbi.nlm.nih.gov/genbank) was used to find the protein sequence. The system value was also set to > 160, and the minimum folding free energy was adjusted under 20 to predict the miRNA target locations. A higher score showed a better capacity to bond. The selection of genes dropped above 3% modification frequency due to the vast number of genes differentially expressed in breast cancer. Additionally, the NCBI database was used in advance to look for the gene sequences connected to Homo sapiens.

### Identification of miRNA targets and creation of the gene-miRNA mRNA network

Mirtarbase databases’ miRNA testing may be used to determine which genes to target (http://mirtarbase.mbc.nctu.edu.tw/php/index.php). Genes discovered by utilizing this database were utilized. To enhance the network diagram’s clarity, it was also validated that the NOX4 gene showed High expression. Utilizing the Cytoscape program (version 3.7.1; available for free at http://www.cytoscape.org/download.php), the NOX4-miRNA mRNA association network was created. In addition to the expression network, genes associated with apoptosis were examined.

### Examination of the Gene Ontology (GO)

The target gene underwent GO enrichment using the GOrilla tool to examine better the biological impact of abnormal expression levels and miRNAs in breast cancer (cbl gorilla. cs. Technion.ac.il). A list of related genes is returned for each GO word, with the best-performing genes listed first. Each gene name is followed by a brief description of the gene and the gene symbol [27].

### B. In-Vitro analyzes

#### Cell Culture

The cells were cultured per ATCC recommendations after receiving them from Iran’s National Cell Collection (Pasteur Institute, Iran). There were used two distinct breast cancer cells. MCF-7 and MDA-MB-231 cells were grown in Dulbecco’s modified Medium containing (DMEM; Gibco) enhanced with 10% FBS (Gibco, USA), 50 U/ml penicillin, and 50 µg/ml streptomycin at 37 °C in 5% CO2 (Sigma-Aldrich, USA). The Control group consisted of regular human breast epithelial cells (HMEC). The human breast epithelial cells (HMEC) were purchased from Iran’s National Cell Collection (Pasteur Institute, Iran).

### DNA Development and Gene Targeting

The NOX4 gene was silenced in human breast carcinoma cell lines using the CRISPR/Cas9 technique. The GenBank sequence database of the National Center for Biotechnology Information contains the NOX4 gene sequence (National Biosciences, Inc., Plymouth, MN). To create single guide RNA (sgRNA) sequences that targeted various regions of the NOX4 gene, the specifically designed CRISPR CHOPCHOP websites (https://chopchop.cbu.uib.no) and (http://crispr.mit.edu/) were utilized. Three vectors were constructed: pX459 (which contained the U6 promoter-sgRNA intubation site-sgRNA scaffold and the CAG promoter-Cas9-T2Apuromycin N-acetyltransferase gene-bovine hormonal polyadenylation signal); pX460-1 (which contained the U6 promoter-sgRNA intubation site-sgRNA scaffold and the CAG promoter-enhanced GFP); sgRNA-encoding oligonucleotides with steaky ends were generated (Macrogen Inc., South Korea), synthesized, phosphorylated, and inserted into BbsI-digested and gel isolated carriers to accomplish this purpose (using Gel Extraction Kit; DENAzist Asia Co., Iran).

In “CRISPR du-HITI” approaches plasmids, the PAM domain was added following the sgRNA coding portion. DsRed2, the herpes simplex virus thymidine kinase polyadenylation signal, the CMV promoter, PuroR, IRES2, EGFP, SV40 polyadenylation transmitter, and the right homologous arm (550 bp) were all present in the plasmid used for “CRISPR HDR” targeting (808 bp). Table 1 demonstrates the plasmids employed to transfect breast malignant cell lines utilizing Lipofectamine 2000 chromophore (Thermo Fisher Scientific, USA). Before being selected by PCR test two weeks after transfection for “CRISPR excision” and eradicating the NOX4 exon, each culture (50 colonies) was monitored to grow.

**Table 1:** The DNA constructs that were employed in this work

Construct	Features	Application
pX459-1	hU6 promoter- sgRNA (downstream of NOX4 Exon1)-sgRNA scaffold-CAG promoter-Cas9-T2A- PuroR-bGH polyA	CRISPR Excision CRISPR du-HITI
pX459_2	hU6 promoter- sgRNA (upstream of NOX4 Exon 1)-sgRNA scaffold-CAG promoter-Cas9-T2A- PuroR-bGH polyA	CRISPR Excision CRISPR du-HITI
pX460_11	hU6 promoter-sgRNA (downstream of NOX4 Exon) plus PAM-sgRNA scaffold-CAG promoter-EGFP-bGH polyA	CRISPR du-HITI
pX461_11	hU6 promoter-sgRNA (downstream of NOX4 Exon) plus PAM-sgRNA scaffold-CAG promoter-PuroR-bGH polyA	CRISPR du-HITI

### DNA extraction and sequencing

Both wild-type and knockout cell lines had their DNA molecules extracted using the DNA-extraction Kit (Cinnacolon, Tehran, Iran), which was then subjected to PCR assay. PCR-amplified results were presented to Sanger Sequencing technology following a smooth reaction recovery (Macrogen Inc., South Korea).

### T7 endonuclease test for mismatched duplex identification

A mismatch-sensitive T7 endonuclease 1 assay (New England Biolabs) was utilized to verify that DNA breakage and specific nucleotide disruption occurred at the designated location. Following the directions provided by the manufacturer, DNA extraction from the colonies using the FavorPrepTM GEL Purification and DNA extraction kit. Each Purified DNA was divided into 10 µl (200 ng), 2 µl (10X NE-Buffer 2) buffer, and 19 µl (nuclease-free water) and placed in separate microtubes. The samples were warmed at 95 °C for 10 min. It was then given time to cool gradually to room temperature. T7 endonuclease I (5 units/ml) was added to 19 µl of each test, which was then incubated at 37 °C for 15 min before being analyzed on an agarose gel. Tanon-electrophoretic software detected band strengths, and the desired disruption was observed.

### Quantitative reverse transcription PCR

Total RNA was extracted from wild-type and knockout cells using the Y-Tizol RNA extraction Kit (Yekta-Tajhiz, Iran). The quality and variety of the collected RNA were assessed using a 2000 Nanodrop spectrometer (Thermo Scientific, USA). 1 µg of total RNA was reverse transcribed, and cDNA was produced using random hexamer primers and MMLV reverse transcriptase (Thermo Fisher Scientific, USA). Premix Ex-Taq (Probe qPCR) master mix, 2 µl of cDNA, 500 nM primers, and 100 nM probe (dual-labelled hybridized probes, 5’FAM-3’BHQ1-labeled for NOX4 and 5’CY5-3’BHQ2 for GAPDH) in a 20 µl reaction medium were used in quantitative RT-PCR experiments (Qiagen, USA). The amplification stages employed were 95 °C for five minutes, followed by 40 cycles of 94 °C for thirty seconds, 62 °C for thirty seconds, and 72 °C for thirty seconds. Sanger sequencing was employed to confirm the PCR results’ identity. By subcloning amplified portions and serial dilution, standard curves were produced. Three PCR experiments were conducted on each quantity, and two real-time observations were made. The cycle threshold (Ct) values were then evaluated to the log of the copy numbers. The following formulas were used to determine efficiency (E) for each qPCR strategy based on the computed gradient of standard curves created using 5-fold serial dilution vector compounds: E = (10–1/slope-1) 100%.

All calibration curves in the investigated range were standard and had high correlation coefficients (R2). The matching calibration graph determined the ratio of NOX4 and GAPDH transcription copies. The outcomes of dividing the amounts of the target gene (NOX4) and the standard gene (GAPDH) for two sets of cDNAs were displayed.

### Sphere formation examination

Breast cancer cells MCF-7 and MDA-MB-231 transfected with plasmids was seeded in six-well plates and cultured (Corning, NY, USA). As previously stated [19], cells (2 × 10^5^) were grown in serum-free DMEM medium with added EGF, hFGF, and penicillin/streptomycin (Gibco). Prototypes of spheroids were fixed, stained with crystal violet dye solution, and then recognized using a light stereomicroscope (Olympus, Tokyo, Japan).

### Analyze the colony growth and proliferation of CCK-8

With a CCK-8 detection kit, the CCK-8 assessment was completed (Dojindo Japan). Before the cell lines received the CCK-8 chemical treatment, the transfected cells were seeded into culture plates and grown for 10 h. 450 nm was used to measure absorbance.

### Transwell invasion screenings

Cancer cells were placed on a pre-coated plate with adherent cells in a 24-well transwell tube (Corning) (BD Biosciences, San Jose, CA, USA). After 24 h of treatment, the top surfaces were brushed, and the invaded regions were fixed with 4% paraformaldehyde and stained with Giemsa. Afterwards, the optical microscope was used to observe the cells.

### Luciferase gene reporter experiment

Luciferase reporter complexes were created using the wild-type and mutant genotypes for the E2F1 connection of the Nanog promoter region. MCF-7 and MDA-MB-231 cells were co-transfected with the vectors and E2F1 using the Lipofectamine 2000 reagent (Thermo Fisher, USA). Promega’s Dual-Luciferase Reporter Assay Kit evaluated the Renilla vector’s performance (Promega).

### MTT analysis

The MTT cytotoxicity Kit I (Roche, Switzerland) was used to verify the cell viability. 5 × 10^3^ cells/well on a 96-well flat-bottomed plate were seeded and cultivated at 37 °C in a 5% CO2 incubator. The samples in each well were washed thoroughly with PBS over three days (24 h, 48 h, and 72 h), during which the cell viability was assessed. 100 µl of serum-free medium and 5 µg/ml Sigma MTT were added to each well, and they were then cultured for 4 h at 37 °C in a CO2 incubator. The media was gradually removed and replaced with DMSO. Using a State Fax-2100 Microplate reader, the optical density at 570 nm was compared to the background at 690 nm.

#### Cell cycle evaluation

The cells were fixed in absolute ethanol for 24 h. Before being labelled for 15 min using PI/RNase staining solution from BD Bioscience Pharmingen, the cells were washed twice in PBS. FACS flow cytometry was performed to ascertain the cell population’s DNA composition. FlowJo V10 software (Tree Star, Ashland, OR) assessed the cell cycle statistics.

### Quantitative real-time PCR analysis

A quantitative real-time PCR method with SYBR green amplification was employed to measure the levels of the proapoptotic genes P57, P21, Prkca, MDM4, and FADD, as well as the antiapoptotic genes BCL2 and SURVIVIN. The manufacturer performed quantitative real-time PCR as directed using an SYBR® Premix Ex TaqTM II kit (TaKaRa, Japan) and particular primers (Table 2). By adjusting the corresponding GAPDH amount, relative genomic levels were measured. The experiments were carried out twice.

**Table 2:** List of specific primers used in this research

Gene	Primer	Sequence 5’----------------------3’	TM(°C)
NOX4	NOX4-F NOX4R	TTGGCTTTGGATTTCTGGAC TGGGTCCACAACAGAAAACA	59
P57	P57-F P57-R	CTTCTTTGACCCTGACACC CTGAACATGGAGAGATAGTGC	59
Survivin	Sur-F Sur-R	GAGAACGAGCCAGACTTGG GCTTTCCTTTCTGTCAAGAAGC	62
BCL2	BCL2-F BCL2-R	TGTGGCCTTCTTTGAGTTCG TACAGTTCCACAAAGGCATCC	58
Prkca	Prkca-F Prkca-R	′CGACTGTCTGTAGAAATCTGG CACCATGGTGCACTCCACGTC	59
MDM4	MDM-F MDM4-R	AATGATGACCTGGAGGACTCTA ACTGCCACTCATCCTCAGAGGTA	59
FADD	FADD-F FADD-R	C GGAAATGGGACAAAACATCCT TGCGGGAGTAGTTGGAAAGT	59
GAPDH	GDH-F GDH-R	GCCAAAAGGGTCATCATCTCTGC GCCAAAAGGGTCATCATCTCTGC	62
E2F1	E2F1-F E2F1-R	TGATTGTGGCAAAGGAGGA CTCTTCCTTGCTCGTTGTTGGTAT	62

#### Statistic assessment

Every study was conducted twice, and the data were presented as mean ± SD. The group variations were examined using one-way ANOVA and independent variables t-tests. The analysis was done with SPSS software, and the graphics were made with Graph-Pad Prism. A P value of less than 0.05 was used to indicate significance.

## Results

### Study findings, selection criteria, and features

**Fig. 1:**
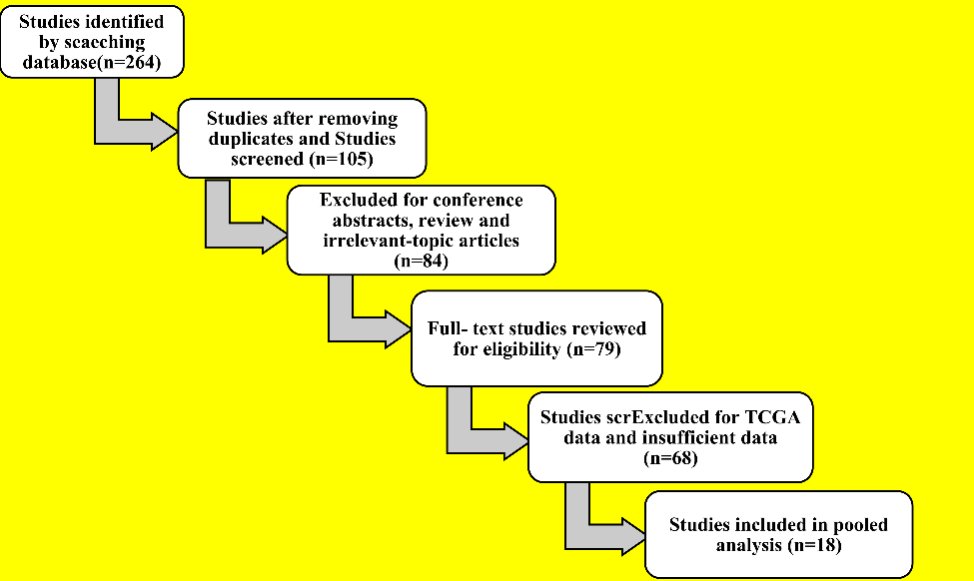
Flow diagram of the bioinformatics study selection

A total of 264 potentially relevant articles were found using online databases. We initially removed 105 duplicates to locate relevant publications. After looking at conference papers, review articles and research that had nothing to do with our study issue, we excluded 84 papers. Then, 79 publications’ whole texts were examined. During this phase, 68 publications were discarded due to insufficient TCGA data. Finally, the 18 publications included in our meta-analysis satisfied our needs well. A flowchart that represents the study recruiting procedure is shown in Fig. 1. All research studies were examined between 2014 and 2021. The study’s qualifying sample size ranged from 25 to 150 individuals. The carcinoma cell lines investigated were MDA-MB-468 (4 samples), MDA-MB-231 (5 samples), MDA-MB-453 (2 samples), and MCF-7 (7 samples). All eligible studies employed RT-qPCR to determine the expression of the NOX4 target sequence. All included papers scored at least six points according to NOS criteria, suggesting they were all of excellent high quality and appropriate for this meta-analysis.

### Overexpression of NOX4 and survival outcomes are correlated

**Fig. 2:**
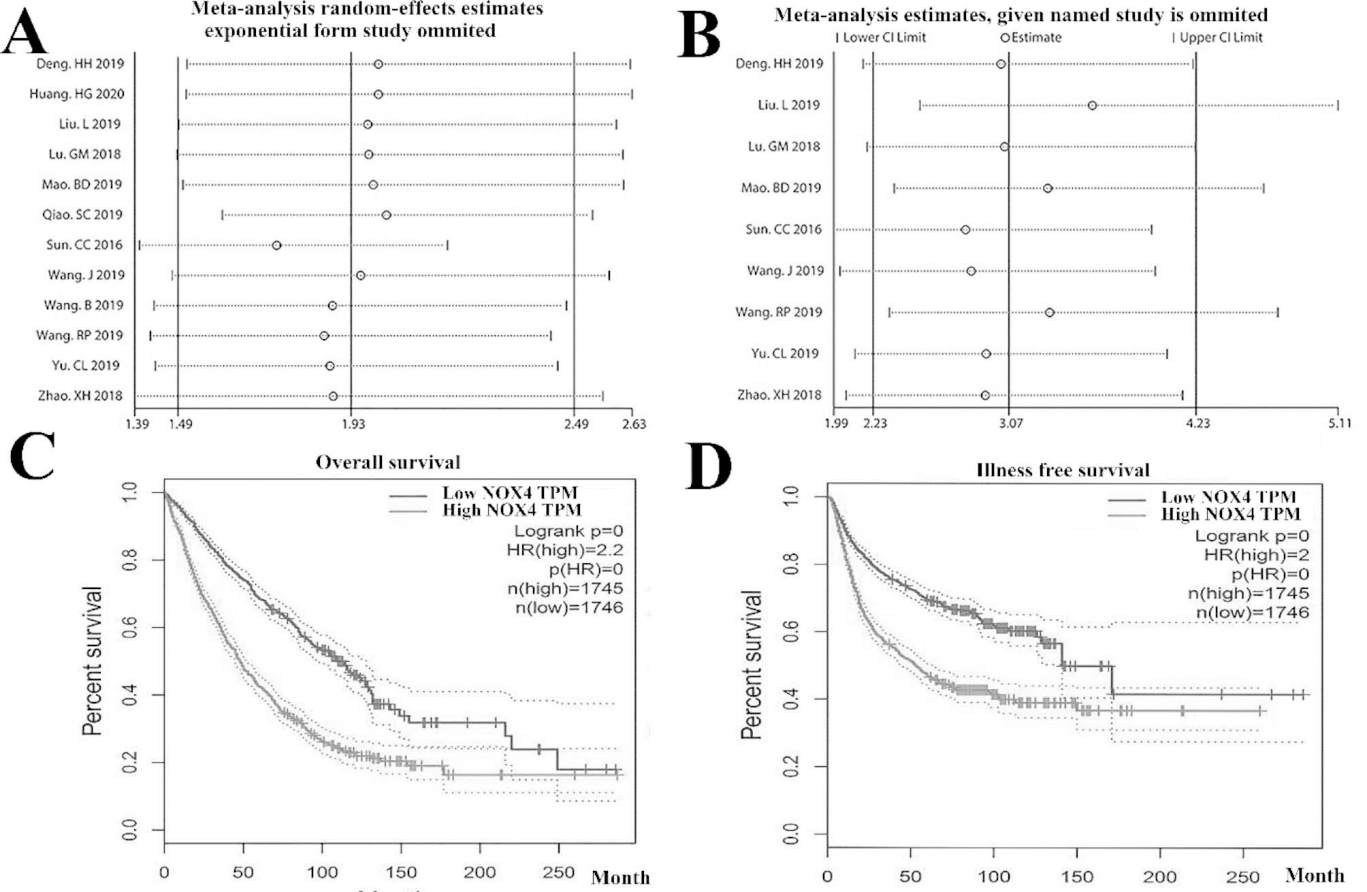
** A** Forest plot illustrating the relationship between NOX4 overexpression and overall survival in breast cancer patients. **B** The forest plot represents the association between lymph node metastases and NOX4 overexpression. **C** NOX4 expression was evaluated for overall survival and **D** disease-free survival using the Kaplan-Meier method in breast cancer patients

In our meta-analysis, we chose 18 publications with a total of 1060 patients who had metastatic cancers to investigate the relationship between NOX4 overexpression and OS. Given the apparent heterogeneity among these experiments (I5 = 83.7%, p < 0.01), the pooled HR and 95% confidence CI for OS were calculated using the random-effect approach (the random effects assumption) [28]. The patient was used for a random effect. A lower likelihood of survival was linked to higher NOX4 expression (1.97, 95% CI 1.27–2.87, P < 0.001) (Fig. 2A). Only two studies have shown a relationship between NOX4 expression and advancement survival; none of these investigations was relevant to DFS. Therefore, we could not conduct a meta-analysis of the predictive significance of NOX4 expression for these survival rates. The entire TCGA dataset, which included 3491 patients partitioned into min and max expression categories based on NOX4 expression, was combined with NOX4 expression information to generate survival plots utilizing GEPIA to validate the results of our meta-analysis and assess the prognostic significance of NOX4 encoding for other survival rates. The results demonstrated that higher NOX4 expression was linked to poorer OS and DFS (Fig. 2B, C), supporting the conclusions of this meta-analysis.

### A meta-analysis looked at the connection between NOX4 overexpression and lymph node metastasis

To determine whether there was a connection between NOX4 expression and lymph node metastases, this meta-analysis looked at seven publications that included 720 cancer patients. Since slight variation existed between these studies (I5 = 33.2%, p = 0.127), we utilized the fixed-effect model to calculate the combined OR and 95% confidence interval for lymph node metastasis. Lymph node metastasis was demonstrated to be more likely when NOX4 expression was greater (OR = 2.93, 95% CI 2.18–4.31, P < 0.001) (Fig. 2D). Since there were only research reports demonstrating a link between NOX4 expression and metastatic illness, it could not conduct research like a meta-analysis to investigate the relationship between NOX4 expression and distant metastasis because of the small sample size.

**Fig. 3:**
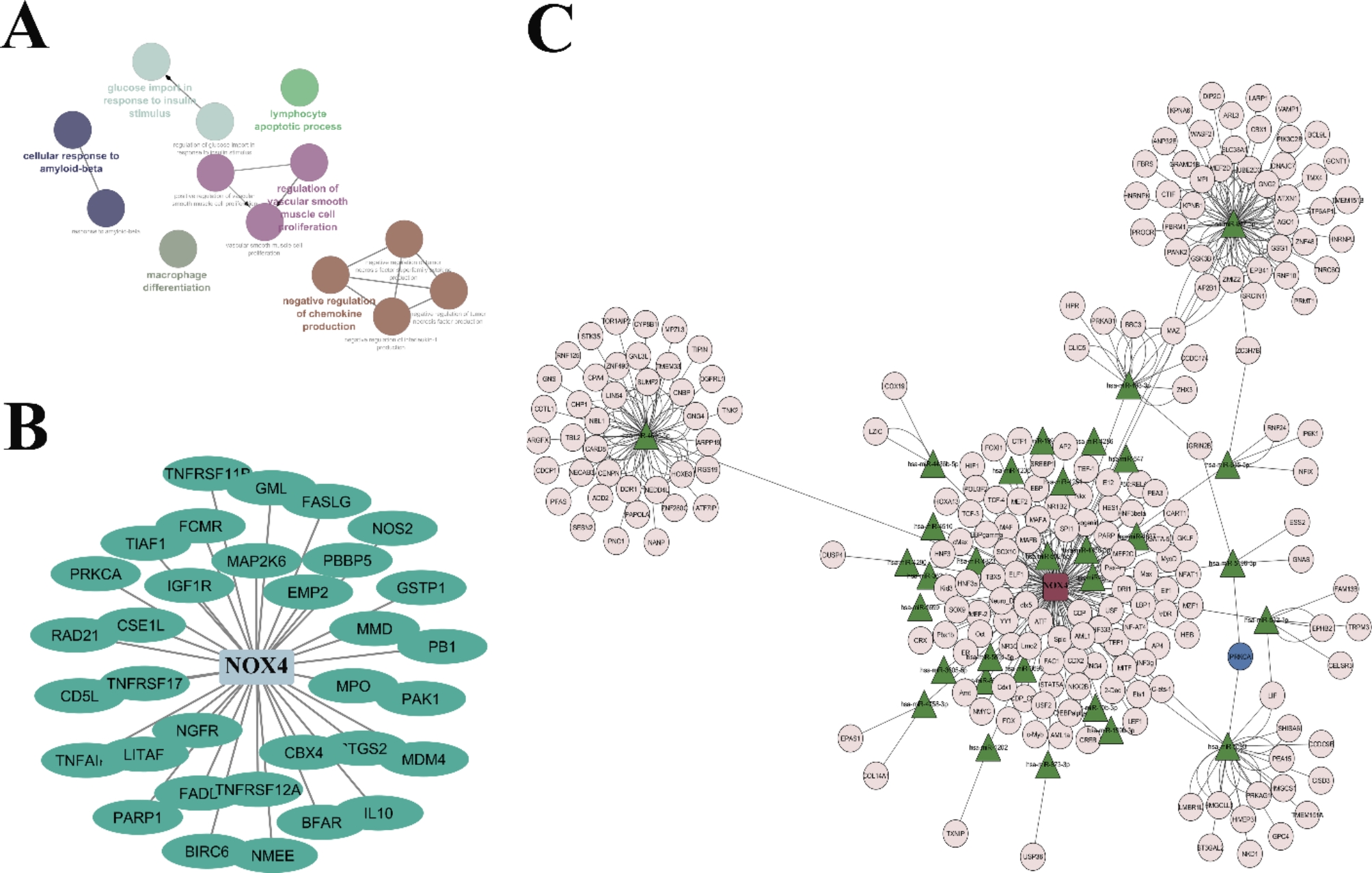
** A** Using clueGo, the apoptotic gene pathway associated with NOX4 is shown. According to the KEGG database, the genes have been enhanced. **B** The QIAGEN dataset displays all genes connected to apoptosis and is correlated with NOX4. **C** For NOX4, the L network is visible. Depending on the L database, all genes connected to the mice discovered for NOX4 have been retrieved and shown. The only gene that appeared in the L network and the expression connection with NOX4 was the prkca gene (marked in blue)

The signaling pathways genes, mutation occurrence, and copy number change (CNV) differ in breast cancer depending on bioinformatics analyses. ClueGo was employed to identify the apoptosis-inducing gene pathways connected to NOX4 in Fig. 3A. All apoptotic genes have been identified using the NOX4 gene and the QIAGEN dataset (Fig. 3B). The network of NOX4 was then introduced. The L database was searched for all NOX4 mouse-related genes, which are displayed in Fig. 3C. In both the L network and the expression association with NOX4, the prkca gene was the sole gene that was present. The network of RNA internal competition for NOX4 was shown.

### B. In-Vitro analysis

#### NOX4 is the target of CRISPR/Cas9-mediated knockdown techniques

**Fig. 4:**
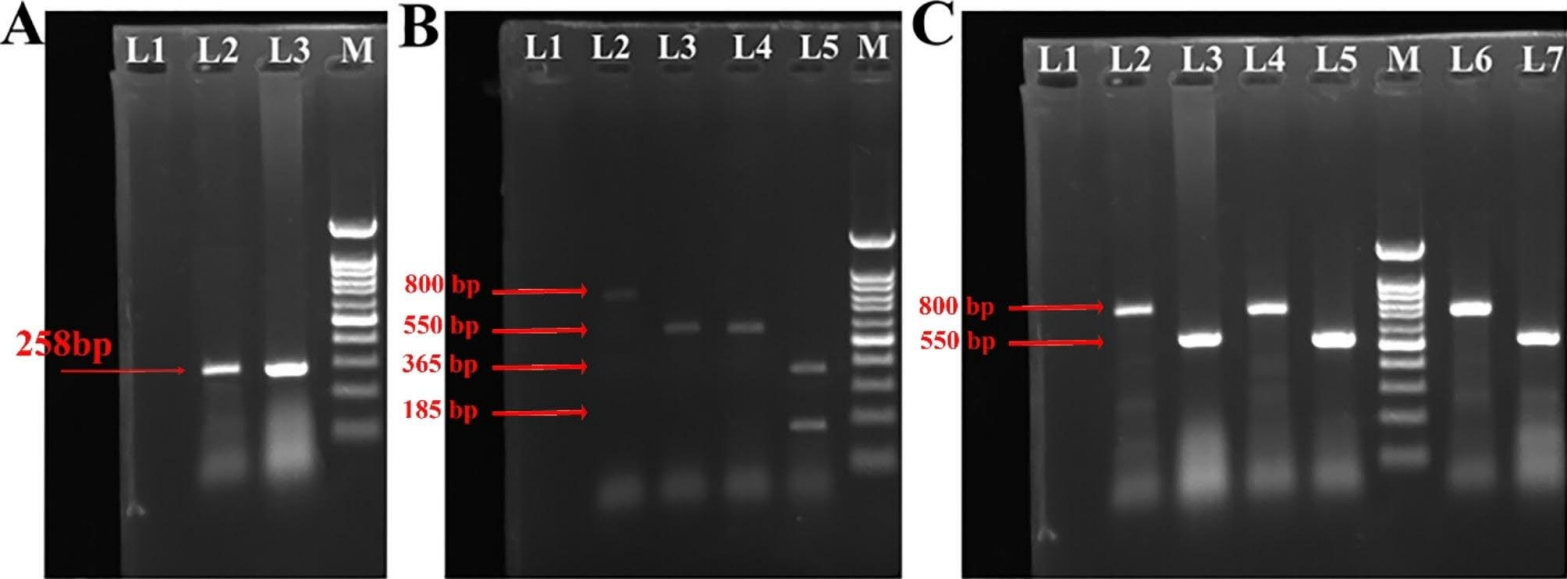
** A** pSpcas9 vectors had 258 bp bands that indicated the existence of sgRNA1 and sgRNA2. The 258 bp bands in L2 and L3 proved that sgRNAs 1 and 2 were present in vector pSpcas9. **B** The PCR result for the target groups (PX459-NOX4-sgRNA1, 2) is 550 bp (L3 and L4), whereas the control (PX459) is 808 bp (L2). L5 is a cleaved mismatch product. **C** various CRISPR techniques using CRISPR-excision (L2 and L3), CRISPR-HDR (L4 and L5), and CRISPR du-HITI (L6 and L7), PCR of wild type (800 bp) and knockout alleles (550 bp) was demonstrated. L1 in all figures is negative PCR control and M is 100 bp DNA ladder

By deleting a section of the NOX4 gene that was crucial for the protein’s conformations or introducing a closed loop signal into the NOX4 locus to encourage early transcription termination, we hoped to disrupt NOX4’s everyday activities. The first method here is called “CRISPR ablation,“ including using two sgRNAs to precisely remove a genomic section. In this approach, we used two sgRNAs to direct Cas9’s endonuclease activity to either side of the NOX4 exon. Following the creation of the pSpcas9-sgRNA 1 and pSpcas9-sgRNA 2 vectors, plasmid amplification was carried out in E. coli top10f. The PCR findings employing pSpcas9-sgRNA 1 and pSpcas9-sgRNA 2 vector-specific primers on microorganisms are shown in Fig. 4A. pSpcas9 vectors had 258 bp bands that indicated the existence of sgRNA1 and sgRNA2. The 258 bp bands in lanes 2 and 3 proved that sgRNAs 1 and 2 were present in vector pSpcas9. A 258 bp section of NOX4 must be removed in light of the effectiveness of PX459-sgRNA 1 and PX459-sgRNA 2 plasmids in the cell lines. The anticipated PCR result for the target groups (PX459-NOX4-sgRNA1, 2) is, therefore, 550 bp, whereas the control (PX459) is 808 bp (Fig. 4B). The presence of a 550-bp band in the PCR results demonstrates the sgRNA’s functional validity (Fig. 4B). In distinct clones created by various CRISPR techniques using CRISPR-excision (Fig. 4C, L2 and L3), CRISPR-HDR (Fig. 4C, L4, L5), and CRISPR du-HITI (Fig. 4C, L6, L7), PCR of wild type (w.t) and knockout alleles was demonstrated.

**Fig. 5:**
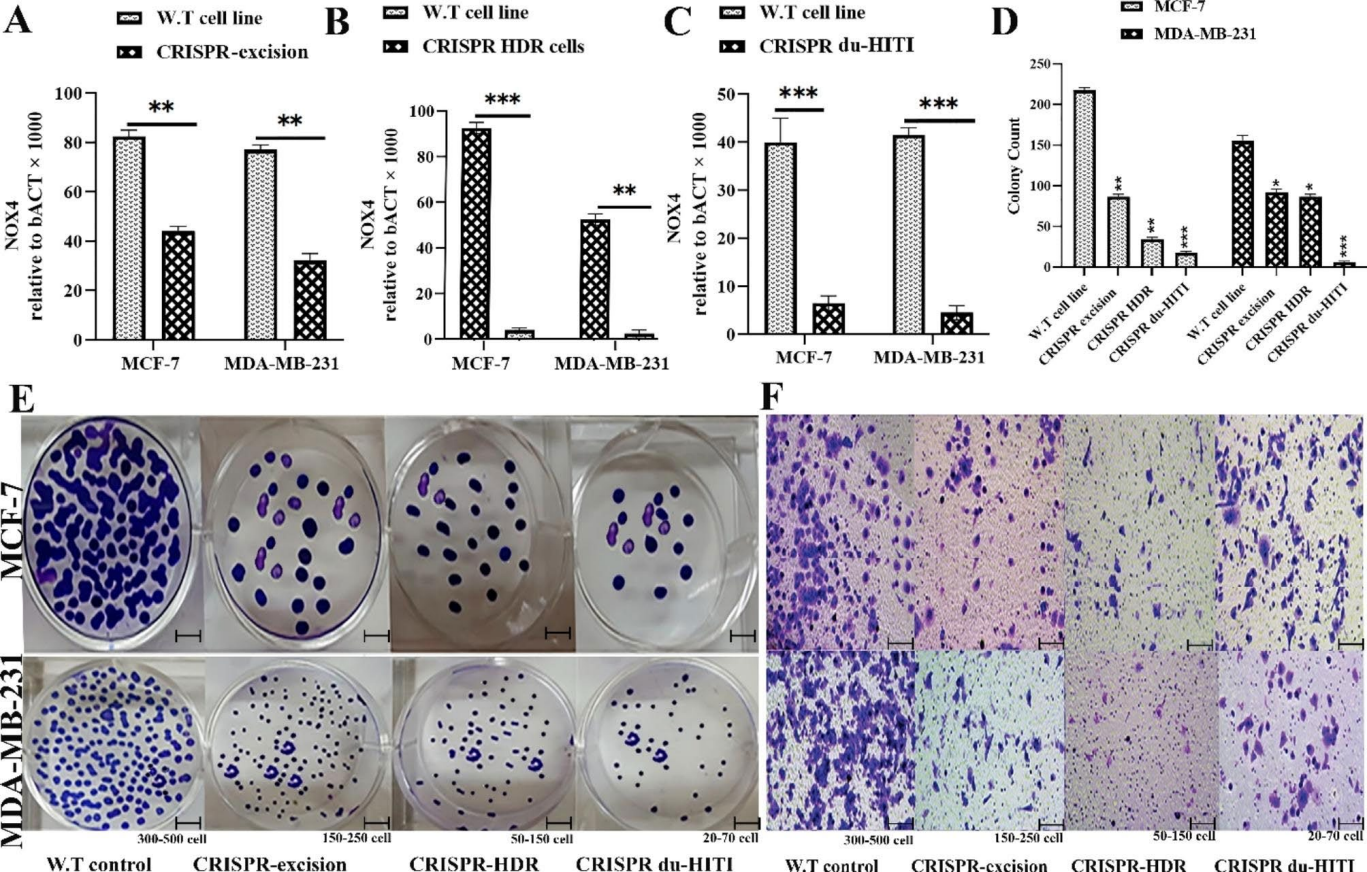
NOX4 is eliminated to impede the culture’s proliferation and invasion of cancerous cells. NOX4 transcript levels were compared to GAPDH levels in MCF-7 and MDA-MB-231 cancer cells that were wild-type, “CRISPR-excision” **(A)**, “CRISPR HDR” **(B)**, and “CRISPR du-HITI” **(C)** knockdown cells. For three samples of cell line cDNAs, the absolute copy numbers for the transcripts NOX4 and GAPDH were calculated using the pertinent standard curves, and the NOX4 and GAPDH transcript amounts were multiplied to display the results. The Mann-Whitney U test examined the statistically significant differences between the wild-type and knockout cell lines. (**D** and **E)** colony formation test on MCF-7 and MDA-MB-231 cells transfected with sh-NOX4 or sh-NC. Sh-NOX4 or sh-NC transfected MCF-7 and MDA-MB-231 cells increased in the CCK-8 assay. **F** The number of MCF-7 and MDA-MB-231 cells that have been invaded. *** p-value < 0.001, ** p-value < 0.01, and * p-value < 0.05

The cell lines used in this investigation were MCF-7 and MDA-MB-231. During the first transfection and screening stage, we obtained 50 MCF-7 clones. Using DNA Template, PCR revealed that ten clones had a copy of NOX4 deleted, but none were homozygous. After transfection and screening, we determined that 2 out of 50 clones were homozygous knockdowns for NOX4, which was verified by PCR analysis of the DNA Sample and sequencing of the PCR findings in CRISPR-excision (Fig. 5A). The second strategy, termed “CRISPR HDR,“ included introducing a reporter gene and a closed-loop indicator in the NOX4 chromosomal area using a straight forward sgRNA and a donor vector with homology arms. We concentrated on the first few exon regions since it was shown that this domain served as a promoter for transcripts originating from exons [29–31]. A transcription termination signal found in the exon ensured that transcripts emanating from its proximal region would be terminated early. Our reporter design included DNA for puromycin dihydrochloride resistance as well as the green fluorescent protein (GFP) locus to identify cells that were sufficiently transformed (Fig. 5B). The “CRISPR du-HITI” method uses two sgRNAs to remove a portion of the chromosome containing the NOX4 exon and insert two reporters and transcription termination indicators using two donor vectors that lack homology arms (Fig. 5C). During the initial transfection, single-cell extraction, and clonal expansion attempt, we successfully obtained eight clones of MCF-7 and 11 MDA-MB-231 cells with sustained green fluorescence profile and puromycin resistance. The knock-in was verified by PCR assays, which also showed the insertion of the necessary fragments. These mutants showed statistically significantly decreased NOX4 expression levels (a 29-fold drop) compared to the control cell line (Fig. 5C).

### Tumorigenic invasion and proliferation are inhibited by NOX4 deletion in vitro

Colony formation test and CCK-8 assay demonstrated that NOX4 suppression prevented MCF-7 and MDA-MB-231 populations from proliferating, whereas NOX4 expression was increased, MCF-7 and MDA-MB-231 cells proliferated (Fig. 5D and E). The transwell invasion experiment research discovered that NOX4 silencing decreased the amount of invading MCF-7 and MDA-MB-231 cell lines; however, it generated the reverse impact when NOX4 expression was increased (Fig. 5F). Therefore, in vitro NOX4 knockdown prevented the growth and invasion of the cancerous MCF-7 and MDA-MB-231 cells. Progesterone and estrogen receptors are expressed by MCF-7 cells, however, none are expressed by MDA-MB-231 cells. The expression of steroid hormone receptors is one of the main subtypes of breast cancer. The level of steroid hormone receptors rises concurrently with the increase in DUOX1, DUOX2, and NOX4 expression in response to 17-estradiol. Because of this, MCF-7 has greater NOX4 expression [29, 32].

#### NOX4 is involved in the maintenance of the breast cancer CSC trait

**Fig. 6:**
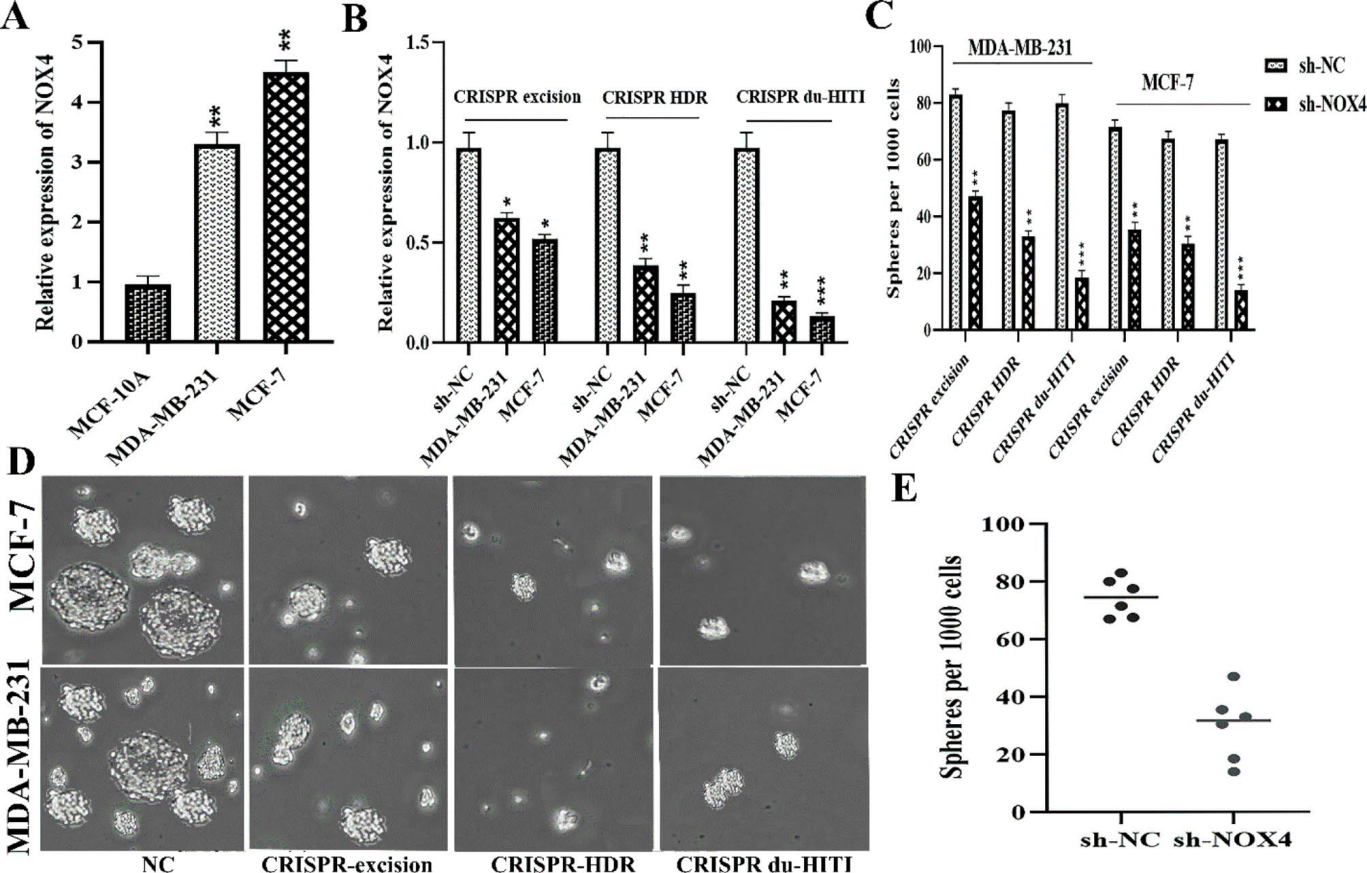
NOX4 plays a part in maintaining the breast cancer CSC characteristic. **A** Breast cancer cell lines MDA-MB-231, MCF-7, and healthy MCF 10 A all expressed NOX4. **B** Breast cancer cells MDA-MB-231 and MCF-7 exhibit NOX4 expression. **C** The mammosphere’s breadth and quantity were reduced when NOX4 shRNA was transfected. **D** and **E** Mammosphere quantity fall after transfection with enhanced NOX4 plasmids. ** P-values l < 0.01, * P-value < 0.05

RT-qPCR analysis revealed that NOX4 function was overexpressed in tumor cells from humans (MDA-MB-231 and MCF-7) as compared to MCF-10 A control lines (Fig. 6A). Using the 3 CRISPR method, shRNA that targeted NOX4 and enhanced expression vectors were added to MDA-MB-231 and MCF-7 human breast cancer cells to reduce or boost expression (Fig. 6B). According to the results of the present investigation, NOX4 silencing reduced the expression of stem factors in MDA-MB-231 and MCF-7 cells. In an inquiry into sphere development, NOX4 suppression reduced the size and frequency of the mammosphere (Fig. 6C, D and E). The results suggested that NOX4 contributed to the maintenance of breast cancer CSC characteristics and that NOX4 was involved in breast cancer cell proliferation and differentiation.

#### E2F1 increased nanog expression at the transcriptional level

**Fig. 7:**
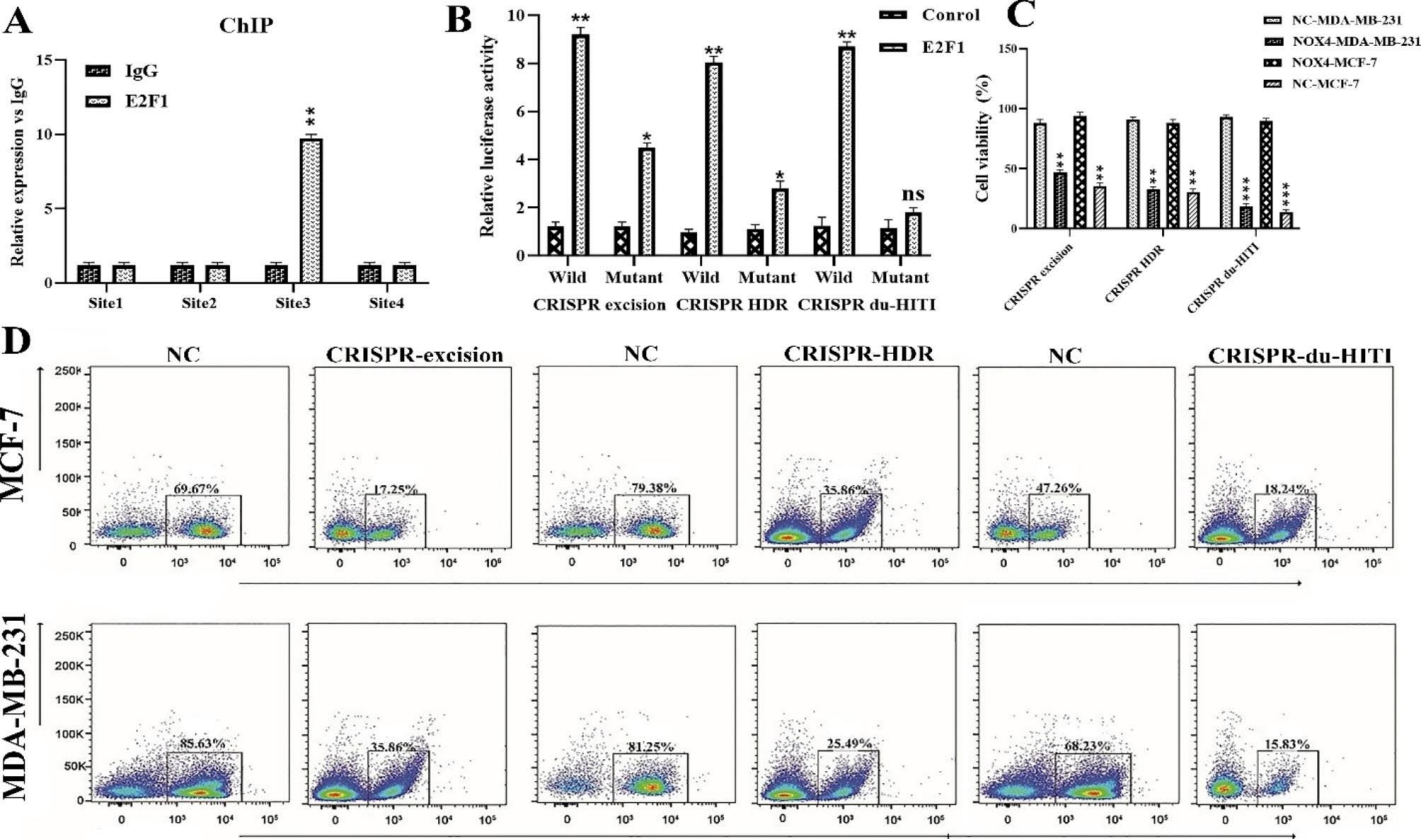
The expression of Nanog was increased by E2F1. **A** The best binding position on the Nanog promoter region was identified using a chromatin immunoprecipitation (ChIP) assay. **B** The creation of the luciferase reporter plasmid (− 586 ~ − 576) and the luciferase activation of wild-type and mutant Nanog promoter region sequences were accomplished. **C** Three separate groups’ growth indices (PI) were examined using a diagram. Compared to the empty control group, PI in the CRISPR du-HITI group was considerably lower (** P < 0.001). **D** By using Annexin V-FITC labeling, the apoptosis of the cells is assessed. Study of the necrotic and apoptotic cells using a flow cytometer. MDA-MB-231 and MCF-7 cells’ apoptotic rates are increased by NOX4 knockout

The promoter of the Nanog gene was separated into four areas (Table 3). A chromatin immunoprecipitation (ChIP) investigation showed that endogenous E2F1 interacts with the Nanog promoter nucleotide sites (− 1000 ~ − 400) (Fig. 7A). Following that, the linkage locations of E2F1 for the promoter Nanog are predicted using bioinformatics approaches. Using PROMO and JASPAR, we found that transcription factor E2F1 might interact with the Nanog gene’s promoter region (+ 100~–2000 nt). Our results showed that the luciferase activity was higher in the non-transgenic variants of Nanog promoter regions than in the recombinant type. This demonstrated that the E2F1 and Nanog promoter regions included the binding site (Fig. 7B).

**Table 3:** Primer sequences for ChIP.

	Sequences 5’-3’
Site 1	forward, 5’-ACCTTCCGCCTGACACCTTTGC-3’ reverse, 5’-TCGGCGGCCTCAACAATGG-3’
Site 2	forward, 5’-TAGATCAGAATAGTCAATGGTGGA-3’ reverse, 5’-AACAAATGTTTTAGCATTGGGATCT-3’
Site 3	forward, 5’-TGTGGCAGAAAGGATTGGA-3’ reverse, 5’-TTGCAGGGTCATCATCAACG-3’
Site 4	forward, 5’-CTCGTACCAGGCGAAAAAAG-3’ reverse, 5’-ACCAGAGGGGACACAGTACA-3’

### NOX4 knockout by CRISPR/Cas9 increased apoptosis rate by suppressing apoptosis-related genes

The impact of NOX4 suppression on MDA-MB-231 and MCF-7 cell proliferation per 72 h was evaluated using MTT. All CRISPR methods reduced the rate of increase of MDA-MB-231, MCF-7 breast cancer cells (P < 0.001), and there was a substantial difference between the growth rates of the control and mutant groups (Fig. 7C). An Annexin V-PI screening procedure was utilized to look at any differences in apoptosis between the experimental groups. The frequencies of early, late, necrotic, and surviving cells are shown in Fig. 7D. Three CRISPR techniques resulted in early, late, and necrotic cell death rates in the blank control cells of less than 9%. Over 91% of the rats in the control groups still had viable cells. The viability for CRISPR excision, CRISPR HDR, and CRISPR du-HITI in MDA-MB-231 cells was 35.86, 25.49, and 15.83%, respectively. In MCF-7 cells, CRISPR excision, CRISPR HDR, and CRISPR du-HITI revealed different viability levels of 17.25, 35.86, and 18.24% (Fig. 7D).

**Fig. 8:**
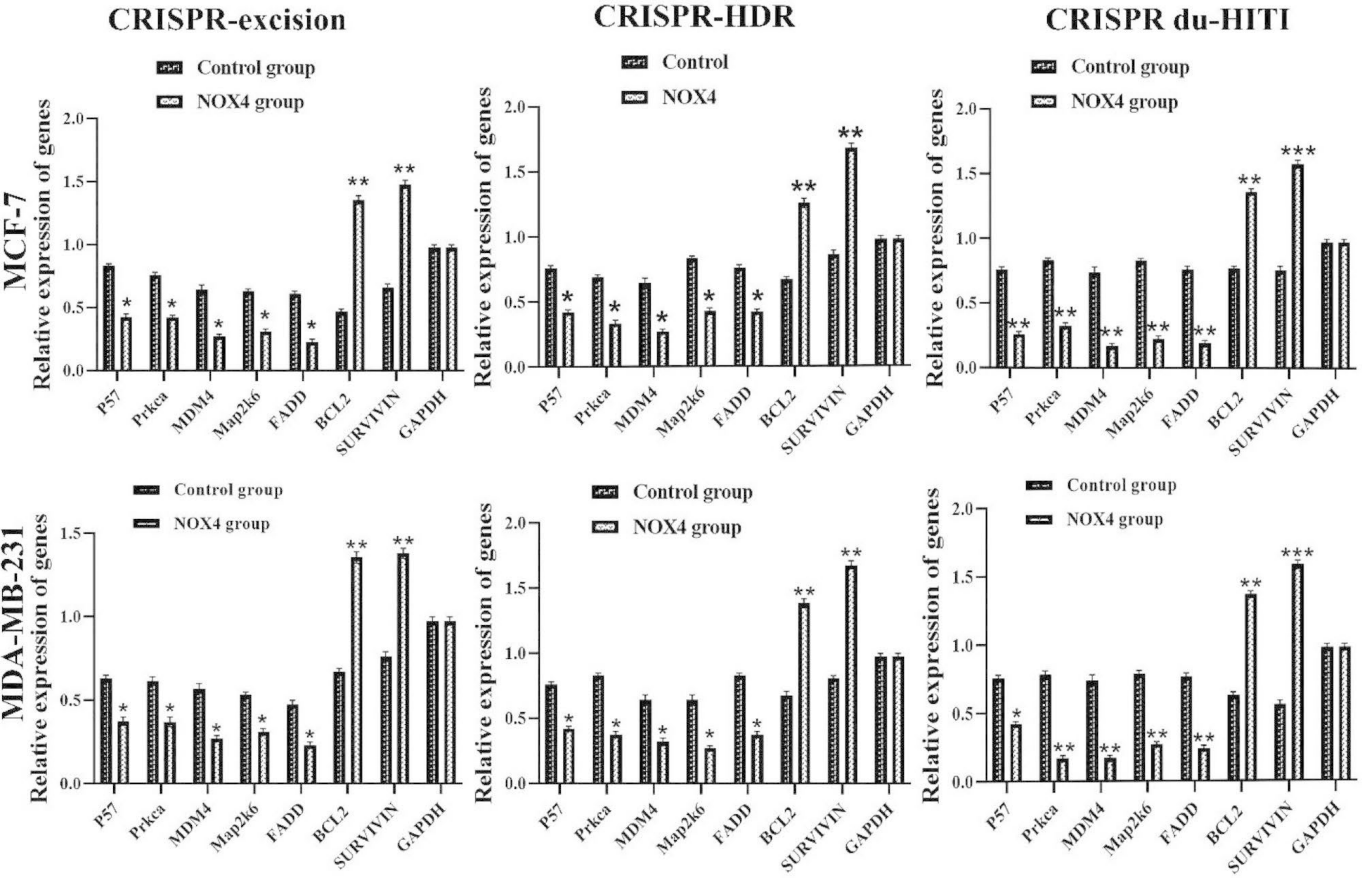
Knockout of NOX4 gene in MDA-MB-231and MCF-7cell line leads to induction of expression of pro-apoptotic genes P57, Prkca, MDM4, Map2k6, and FADD at a significant level ** P < 0.01 and reduction of expression of anti-apoptotic genes SURVIVIN and BCL2 at a significant level *** P < 0.001. The GAPDH reference gene normalized data. There was no significant difference in the expression of pro/anti-apoptotic genes in the Blank control groups

Accelerated cell proliferation was related to cell cycle progression. The control of the cell cycle in the CRISPR excision, CRISPR HDR, and CRISPR du-HITI cell types was investigated using flow cytometry. Compared to the control groups, NOX4 knockout cells had higher G0.G1 and lowered S phase to G2.M phase ratios. These results showed that NOX4 knockdown impaired cell cycle progression. For the current investigation, the expression of the apoptosis-inducing genes P57, P21, Prkca, MDM4, Map2k6, and FADD and the antiapoptotic genes BCL2 and SURVIVIN, was determined using real-time PCR in two cell lines. Apoptosis-inducing gene expression was considerably higher in the ablation groups than in the blank control group (Fig. 8, p < 0.01). After that, we looked at the expression of two different cell types’ BCL2 and SURVIVIN antiapoptotic genes. BCL2 and SURVIVIN gene expression was higher in the control cells than in the knockout cells (p < 0.05).

## Discussion

Numerous tumors showed downregulation of NOX4, associated with metastasis and survival in human malignancies [25, 26, 33]. However, the clinical relevance of NOX4 upregulation in human malignancies was still up for discussion. As a result, we conducted a comprehensive investigation of the diagnostic value of NOX4 overexpression using data from The Cancer Genome Atlas (TCGA) and computational studies. We thoroughly investigated the biological functions of NOX4 in cancerous tumors and the associated processes. This study found that increased NOX4 expression was associated with worse OS and DFS and an increased incidence of lymph node metastases. The intricate mechanisms underlying NOX4’s carcinogenic actions may help to explain why NOX4 increased expression in malignancies was clinically significant [34]. By sponging miRNAs and altering the synthesis of their targets, including miR-185-3p/E2 F1/Nanog [35], miR-625/CCND1 [36], miR-524-5p/YB1/ZEB1 [37], miR-124-3p/EZH2 [38], and miR-765/APE1 [39], the majority of researchers discovered that NOX4 could increase malignant cells’ aggressiveness. This study is a meta-analysis, and the main limitations of a meta-analysis are that it combines different types of studies and that the summary effect may ignore important differences between studies.

The findings of this study showed that breast carcinoma cell lines produced more NOX4, which may be associated with a poorer prognosis. These results suggested that NOX4 was a regulator of breast cancer. It investigated how NOX4 affected breast carcinoma cells’ biological morphology and stemness. Gain and loss-of-functional studies showed that NOX4 accelerated sphere growth, produced more stem factors, and helped maintain the features of breast cancer CSCs, indicating that NOX4 was involved in malignant cell differentiation [40]. Breast, lung, colorectal, malignant, and glioblastoma were previously linked to CSCs [37]. Important biological properties of cancer that cause invasion and metastasis. Various investigations [41, 42] linked CSCs to tumor metastasis and invasion. Researchers conducted experiments to learn more about how NOX4 regulated the aetiology of breast cancer. E2F1 connected with the promoter of the Nanog gene, demonstrating that it served as a transcriptional activator. A luciferase reporter assay, ChIP, was used to validate the proactive approach of E2F1 and Nanog while establishing the NOX4/E2F1/Nanog regulatory circuit. The transcription factor E2F1 has been identified as an oncogenic element in colorectal and bladder cancer [43, 44]. Additionally, it was shown that E2F1 was up-regulated and active in the carcinogenesis of breast cancer, contributing to the creation of Nanog in this disease. The importance of functional studies in defining proteins’ biological roles increased as more molecules were found. Similar to how protein-coding genes were studied, approaches meant to limit the production of proteins were crucial for comprehending the biological functions of NOX4. However, two frequently utilised strategies, RNA interference and antisense methods proved ineffectual for molecules with nucleus localization [45]. These strategies are also connected to insufficient target gene suppression, off-target consequences, and technical variations. A fantastic possibility was employing recently developed genome editing technology to move a gene’s chromosomal position so that the associated NOX4 became inactive [46].

Furthermore, changes in the related genomic areas must be significant enough to negatively affect the growth and biological operation of the synthesized RNA. For instance, a direct excision followed by NHEJ could not sufficiently modify a particular gene’s structural makeup [47–52]. Three different CRISPR-mediated KO methods, including “CRISPR excision,“ “CRISPR HDR,“ and “CRISPR du-HITI,“ were used to change the position of NOX4 in several breast cancer cell lines. As previously described, two sgRNAs were used to delete a genomic area of genes [41–29]. The genome contained no reporter sequences since no donor plasmids were used to apply this approach. Selecting Knockout cells thus took much time. On the other hand, “CRISPR HDR” uses donor plasmids, enabling the user to select the molecular pathways depending on fluorescence and antibiotic resistance [50–54].

However, homology components must be cloned into the donor vector for this method to function. Cancer cells might be chosen using the “CRISPR du-HITI” technique, which does not need the presence of homologous bands. Using this technique, we managed the generation of Knockouts so that each cell had antibiotic resistance and fluorescence traits that made it simple to recognize a double allele Knockout. The NOX4 Knockout cells generated in this study were verified using classical PCR, sequencing, and RT-qPCR. Because the qPCR forward and reverse primers were equal to an exon, we did not anticipate any stimulation results from “CRISPR excision” and “CRISPR du-HITI” Knockout cells. Each of these processes included the deletion of an Exon. The initial part of the exon was labelled with a reporter/transcription termination signal-containing component in “CRISPR HDR,“ nonetheless. An amplifying outcome was therefore predicted. On the NOX4 Knockout cells, RNA-seq, a soft agar colony formation test, and MTT studies were carried out. In these cells, many genes were expressed in various ways. MTT and soft agar colony growth confirmed the decreased proliferative capacity of the NOX4 Knockout cells. This outcome aligned with earlier findings that NOX4 inhibition affected the genes controlling cell proliferation.

## Conclusion

In summary, we discussed three CRISPR/Cas9-mediated methods that helped evaluate the functioning of lncRNAs. Furthermore, the NOX4 Knockout cell lines created in this research might be used for additional analytical studies to reveal the complete spectrum of NOX4 capabilities. It was easy to use and could create homozygous individuals who were knockout for a specific gene of concern using the du-HITI method described in this work.

### Electronic supplementary material

Below is the link to the electronic supplementary material.

Supplementary Material 1

## Abbreviations

KO: Knockouts
HR: Hazard ratio
CI: Confidence interval
OR: Odd ratio
CSCs: Cancer stem cells
ROS: Reactive oxygen species
CRI: Clustered Regularly Interspaced
CRISPRi: CRISPR-mediated inhibition
TALENs: Transcription activator-like effector nucleases
ZFNs: Zinc-finger nucleases
DSBs: Double-strand breaks
OS: Overall survival
DFS: Disease-free survival

## References

[1] Verras GI, Tchabashvili L, Chlorogiannis DD, Mulita F, Argentou MI. Updated clinical evidence on the role of adipokines and breast cancer: a review. Cancers. 2023 Mar 3;15(5):1572. 10.3390/cancers15051572 PMC10000674 36900364

[2] Miricescu D, Totan A, Stanescu-Spinu II, Badoiu SC, Stefani C, Greabu M (2021). PI3K/AKT/mTOR signaling pathway in breast cancer: from molecular landscape to clinical aspects. Int J Mol Sci.

[3] Hamer J, Jones E, Chan A, Tahmasebi F. Can we routinely employ the use of low-pressure Gynaecological Laparoscopy? A systematic review. Cureus. 2021;13(5). 10.7759/cureus.15348 PMC8244579 34235025

[4] Umeda M, Ikeuchi M, Ishikawa M, Ito T, Nishihama R, Kyozuka J, Torii KU, Satake A, Goshima G, Sakakibara H (2021). Plant stem cell research is uncovering the secrets of longevity and persistent growth. Plant J.

[5] Valle S, Martin-Hijano L, Alcalá S, Alonso-Nocelo M, Sainz B (2018). The ever-evolving concept of the cancer stem cell in pancreatic cancer. Cancers.

[6] Huang Y, Mo W, Ding X, Ding Y. Long non-coding RNAs in breast cancer stem cells. Med Oncol. 2023 May;13(6):177. 10.1007/s12032-023-02046-1 37178429

[7] Krylatov AV, Maslov LN, Voronkov NS, Boshchenko AA, Popov SV, Gomez L (2018). Reactive oxygen species as intracellular signaling molecules in the cardiovascular system. Curr Cardiol Rev.

[8] Maraldi T, Angeloni C, Prata C, Hrelia S (2021). NADPH oxidases: Redox regulators of stem cell fate and function. Antioxidants.

[9] Cho SY, Kim S, Son MJ, Kim G, Singh P, Kim HN (2019). Dual oxidase 1 and NADPH oxidase 2 exert favorable effects in cervical cancer patients by activating immune response. BMC Cancer.

[10] Moghadam ZM, Henneke P, Kolter J (2021). From flies to men: ROS and the NADPH oxidase in phagocytes. Front Cell Dev Biol.

[11] Duan J, Gao S, Tu S, Lenahan C, Shao A, Sheng J (2021). Pathophysiology and therapeutic potential of NADPH oxidases in ischemic stroke-induced oxidative stress. Oxid Med Cell Longev.

[12] Meitzler JL, Makhlouf HR, Antony S, Wu Y, Butcher D, Jiang G (2017). Decoding NADPH oxidase 4 expressions in human tumors. Redox Biol.

[13] Xue L, Li J, Lin Y, Liu D, Yang Q, Jian J, Peng J (2021). m6A transferase METTL3-induced lncRNA ABHD11‐AS1 promotes the Warburg effect of non‐small‐cell lung cancer. J Cell Physiol.

[14] Zare K, Shademan M, Ghahramani Seno MM, Dehghani H (2018). CRISPR/Cas9 knockout strategies to ablate CCAT1 lncRNA gene in cancer cells. Biol procedures online.

[15] Goyal A, Myacheva K, Groß M, Klingenberg M, Duran Arqué B, Diederichs S (2017). Challenges of CRISPR/Cas9 applications for long non-coding RNA genes. Nucleic Acids Res.

[16] Sun C, Li S, Zhang F, Xi Y, Wang L, Bi Y, Li D (2016). Long non-coding RNA NEAT1 promotes non-small cell lung cancer progression through regulation of mir-377-3p-E2F3 pathway. Oncotarget.

[17] Bassett AR, Akhtar A, Barlow DP, Bird AP, Brockdorff N, Duboule D, Ephrussi A, Ferguson-Smith AC, Gingeras TR, Haerty W, Higgs DR (2014). Science forum: considerations when investigating lncRNA function in vivo. elife.

[18] Boettcher M, McManus MT (2015). Choosing the right tool for the job: RNAi, TALEN, or CRISPR. Mol Cell.

[19] Bendixen L, Jensen TI, Bak RO. CRISPR/Cas-mediated transcriptional modulation: the therapeutic promises of CRISPRa and CRISPRi. Mol Ther. 2023 Mar 24. 10.1016/j.ymthe.2023.03.024 PMC10362391 36964659

[20] Sun CC, Li SJ, Li G, Hua RX, Zhou XH, Li DJ (2016). Long intergenic noncoding RNA 00511 acts as an oncogene in non–small-cell lung cancer by binding to EZH2 and suppressing p57. Mol Therapy-Nucleic Acids.

[21] Zhao X, Liu Y, Li Z, Zheng S, Wang Z, Li W, Bi Z, Li L, Jiang Y, Luo Y, Lin Q (2018). Linc00511 acts as a competing endogenous RNA to regulate VEGFA expression through sponging hsa-miR‐29b‐3p in pancreatic ductal adenocarcinoma. J Cell Mol Med.

[22] Sun CC, Li SJ, Li G, Hua RX, Zhou XH, Li DJ (2016). Long intergenic noncoding RNA 00511 acts as an oncogene in non–small-cell lung cancer by binding to EZH2 and suppressing p57. Mol Therapy-Nucleic Acids.

[23] Ding J, Yang C, Yang S (2018). LINC 00511 interacts with miR-765 and modulates tongue squamous cell carcinoma progression by targeting LAMC 2. J Oral Pathol Med.

[24] Kwon Y, Lemieux M, McTavish J, Wathen N. Identifying and removing duplicate records from systematic review searches. J Med Libr Association: JMLA. 2015 Oct;103(4):184. 10.3163/1536-5050.103.4.004 PMC4613377 26512216

[25] Fan Y, Chen J (2017). Clinicopathological significance of survivin expression in patients with cervical cancer: a systematic meta-analysis. Bioengineered.

[26] Hu Y, Zhang Y, Gao J, Lian X, Wang Y (2020). The clinicopathological and prognostic value of CD44 expression in bladder cancer: a study based on meta-analysis and TCGA data. Bioengineered.

[27] Kanehisa M, Furumichi M, Sato Y, Kawashima M, Ishiguro-Watanabe M. KEGG for taxonomy-based analysis of pathways and genomes. Nucleic acids research. 2023 Jan 6;51(D1):D587-92. 10.1093/nar/gkac963 PMC9825424 36300620

[28] Hemilä H. Random-effects assumption in meta-analyses. JAMA. 2019 Jul;2(1):81. 10.1001/jama.2019.5439 31265091

[29] Huang B (2014). Differential expression of estrogen receptor α, β1, and β2 in lobular and ductal breast cancer. Proc Natl Acad Sci.

[30] Prior KK, Leisegang MS, Josipovic I, Löwe O, Shah AM, Weissmann N, Schröder K, Brandes RP. CRISPR/Cas9-mediated knockout of p22phox leads to loss of Nox1 and Nox4, but not Nox5 activity. Redox biology. 2016 Oct 1;9:287 – 95. 10.1016/j.redox.2016.08.013 PMC5021817 27614387

[31] Jafari N, Kim H, Park R, Li L, Jang M, Morris AJ, Park J, Huang C. CRISPR-Cas9 mediated NOX4 knockout inhibits cell proliferation and invasion in HeLa cells. PloS one. 2017 Jan 18;12(1):e0170327. 10.1371/journal.pone.0170327 PMC5242459 28099519

[32] Fabbro D (1986). Epidermal growth factor binding and protein kinase C activities in human breast cancer cell lines: possible quantitative relationship. Cancer Res.

[33] Kalhori MR, Khodayari H, Khodayari S, Vesovic M, Jackson G, Farzaei MH, Bishayee A (2021). Regulation of long non-coding RNAs by plant secondary metabolites: a novel anticancer therapeutic approach. Cancers.

[34] Ding J, Cao J, Chen Z, He Z (2020). The role of long intergenic noncoding RNA 00511 in malignant tumors: a meta-analysis, database validation and review. Bioengineered.

[35] Lu G, Li Y, Ma Y, Lu J, Chen Y, Jiang Q, Qin Q, Zhao L, Huang Q, Luo Z, Huang S (2018). Long noncoding RNA LINC00511 contributes to breast cancer tumourigenesis and stemness by inducing the miR-185-3p/E2F1/Nanog axis. J experimental Clin cancer Res.

[36] Chen Z, Wu H, Zhang Z, Li G, Liu B (2019). LINC00511 accelerated the process of gastric cancer by targeting miR-625-5p/NFIX axis. Cancer Cell Int.

[37] Du X, Tu Y, Liu S, Zhao P, Bao Z, Li C, Li J, Pan M, Ji J (2020). LINC00511 contributes to glioblastoma tumorigenesis and epithelial-mesenchymal transition via LINC00511/miR‐524‐5p/YB1/ZEB1 positive feedback loop. J Cell Mol Med.

[38] Huang HG, Tang XL, Huang XS, Zhou L, Hao YG, Zheng YF (2020). Long noncoding RNA LINC00511 promoted cell proliferation and invasion via regulating miR-124-3p/EZH2 pathway in gastric cancer. Eur Rev Med Pharmacol Sci.

[39] Yan L, Wu X, Liu Y, Xian W (2019). LncRNA Linc00511 promotes osteosarcoma cell proliferation and migration through sponging miR-765. J Cell Biochem.

[40] Li P, Feng C, Chen H, Jiang Y, Cao F, Liu J, Liu P (2018). Elevated CRB 3 expression suppresses breast cancer stemness by inhibiting β-catenin signalling to restore tamoxifen sensitivity. J Cell Mol Med.

[41] Lakota J (2018). Fate of human mesenchymal stem cells (MSC s) in humans and rodents—Is the current paradigm obtained on rodents applicable to humans?. J Cell Mol Med (Online).

[42] Yang S, Dong F, Li D, Sun H, Wu B, Sun T, Wang Y, Shen P, Ji F, Zhou D (2017). Persistent distention of colon damages interstitial cells of Cajal through Ca2+-ERK‐AP‐1‐miR‐34c‐SCF deregulation. J Cell Mol Med.

[43] Su F, He W, Chen C, Liu M, Liu H, Xue F, Bi J, Xu D, Zhao Y, Huang J, Lin T (2018). The long non-coding RNA FOXD2-AS1 promotes bladder cancer progression and recurrence through a positive feedback loop with akt and E2F1. Cell Death Dis.

[44] Su F, He W, Chen C, Liu M, Liu H, Xue F, Bi J, Xu D, Zhao Y, Huang J, Lin T (2018). The long non-coding RNA FOXD2-AS1 promotes bladder cancer progression and recurrence through a positive feedback loop with akt and E2F1. Cell Death Dis.

[45] Fang Y, Fullwood MJ (2016). Roles, functions, and mechanisms of long non-coding RNAs in cancer. Genom Proteom Bioinform.

[46] Awwad DA (2019). Beyond classic editing: innovative CRISPR approaches for functional studies of long non-coding RNA. Biology Methods and Protocols.

[47] Sánchez-Rivera FJ, Jacks T (2015). Applications of the CRISPR–Cas9 system in cancer biology. Nat Rev Cancer.

[48] Han J, Zhang J, Chen L, Shen B, Zhou J, Hu B, Du Y, Tate PH, Huang X, Zhang W (2014). Efficient in vivo deletion of a large imprinted lncRNA by CRISPR/Cas9. RNA Biol.

[49] Paralkar VR, Taborda CC, Huang P, Yao Y, Kossenkov AV, Prasad R, Luan J, Davies JO, Hughes JR, Hardison RC, Blobel GA (2016). Unlinking a lncRNA from its associated cis element. Mol Cell.

[50] Yin Y, Yan P, Lu J, Song G, Zhu Y, Li Z, Zhao Y, Shen B, Huang X, Zhu H, Orkin SH (2015). Opposing roles for the lncRNA haunt and its genomic locus in regulating HOXA gene activation during embryonic stem cell differentiation. Cell Stem Cell.

[51] Zhang E, Han L, Yin D, He X, Hong L, Si X, Qiu M, Xu T, De W, Xu L, Shu Y (2017). H3K27 acetylation activated-long non-coding RNA CCAT1 affects cell proliferation and migration by regulating SPRY4 and HOXB13 expression in esophageal squamous cell carcinoma. Nucleic Acids Res.

[52] Piri-Gharaghie T, Doosti A, Mirzaei SA. Identification of antigenic Properties of Acinetobacter baumannii Proteins as Novel putative vaccine candidates using reverse Vaccinology Approach. Appl Biochem Biotechnol. 2022:1–23. 10.1007/s12010-022-03995-5 35670904

[53] Piri-Gharaghie T (2021). Polycystic ovary syndrome and genetic factors influencing its development: a review article. Personalized Med J.

[54] Azadbakht N, Doosti A, Jami MS. CRISPR/Cas9-mediated LINC00511 knockout strategies, increased apoptosis of breast cancer cells via suppressing antiapoptotic genes. Biological procedures online. 2022 Dec;24(1):1–5. 10.1186/s12575-022-00171-1 PMC9254607 35790898

