# Supplementary material for: Genome editing approaches with CRISPR/Cas9: the association of NOX4 expression in breast cancer patients and effectiveness evaluation of different strategies of CRISPR/Cas9 to knockout Nox4 in cancer cells
Source: BMC Cancer. 2023 Nov 27;23:1155. doi: 10.1186/s12885-023-11183-9 (PMC10683234; doi:10.1186/s12885-023-11183-9)
Supplement: Supplementary file 1 — Supplementary Material 1 [file 12885_2023_11183_MOESM1_ESM.docx]

**Supplementary Material**

# CLASSIFICATION TECHNIQUES

In this subsection, different classification techniques such as K-Nearest Neighbors (K-NN) Altman (1992),

Naive Bayes Classifier (NB) George and Langley (1995), Support Vector Machine (SVM) Cortes and Vapnik (1995), Decision Tree (DT) Quinlan (1986) and Random Forest (RF) Breiman (2005) are discussed.

Among various classifiers, K-Nearest Neighbors (K-NN) Altman (1992) classifier is a simple nonparametric classification method, where a new point is labelled with a label of the majority of its *K* neighbors. The parameter *K* is advised to be odd, since it helps in breaking ties.

Naive Bayes (NB) George and Langley (1995) is statistical classifier which uses Bayes’ theorem to model a relationship between the attributes and class label. Let **x_i_** = (*x_i_*_1_*,x_i_*_2_*,...,x_iD_*) denote features of an observation vector **x** ∈ R*^D^*. Bayes’ theorem states the conditional probability between class label *y* and features
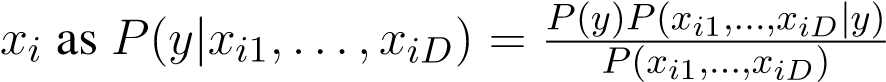
 . NB makes a strong assumption on independence of the attributes, which allows to factorize a term in enumerator as
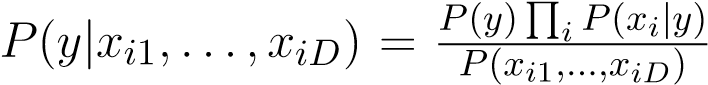
.

Predicted class label is the one that maximizes *P*(*y*|*x_i_*_1_*,...,x_iD_*) given the input data.

Support Vector Machine (SVM) Cortes and Vapnik (1995) is another popular classifier which performs basically for two-class classification by finding a separating hyperplane {**x** ∈ R*^D^* : **w***^T^***x** + *b* = 0}, which has the highest distance from datapoints of both classes, where **w** ∈ R*^D^*, and *b* ∈ R. Extensions to multiclass classification include training one-against-all classifiers for each class separately, or one-against-one for each pair of classes Hsu and Lin (2002). SVM can be extended to non-linear hyperplanes through application of the kernel trick, transforming the data to another space with a kernel function.

Decision Tree (DT) is widely used classifier, where a node corresponds to an input attribute and a set of discrete values, or a threshold splitting the range of possible values in case of continuous variables. Classification trees assign labels to data samples. A new sample is being passed from a root down to a leaf, and leafs hold class labels. Commonly, classification trees are constructed greedily top to bottom, where in every node a variable is chosen along with a split criterion. In the ID3 algorithm Quinlan (1986), the variable with the lowest entropy is greedily chosen as the next node variable. The C4.5 algorithm Quinlan (1993) extends it by, e.g., handling discrete attributes, missing values, or pruning the tree after creation.

On the other hand, Random Forest (RF) Breiman (2005) classifier, consists of a collection of decision trees. Their predictions are combined through majority voting
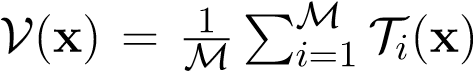
, where each T*_i_* denotes the prediction of a separate decision tree classifier. Those individual trees are learned through bootstrap aggregating, that is selecting with replacement a random sample from the data, and then fitting a classifier to the selected sample.

# FEATURE SELECTION TECHNIQUES

The applied filter feature selection methods are based on MI, which is a measure of mutual dependence of two random variables given as


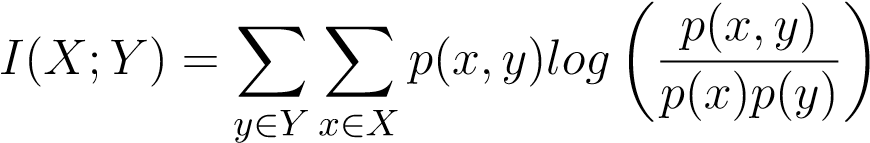
*,*

where *p*(*x,y*) is the joint pdf of *X* and *Y* , and *p*(*x*)*,p*(*y*) are the marginals. The higher the MI value, the

larger the dependence. Other useful relations concerning MI are

*I*(*X*;*Y* ) = *H*(*X*) − *H*(*X*|*Y* )*,*

*I*(*X*;*Y* ) = *KL*(*p*(*x,y*)||*p*(*x*)*p*(*y*))*,*

where *H*() denotes entropy, and *KL*() Kullback-Leibler divergence. Brown et al. Brown et al. (2012a) provide a comprehensive review of those and other methods along with implementations gathered together in the FEAST framework. The Conditional Mutual Information Maximisation (CMIM) Fleuret (2004) method comes as a compromise between the power of the feature alone, and independence of the feature given already selected ones.

The exact formula for CMIM is

*v*(1) = argmax
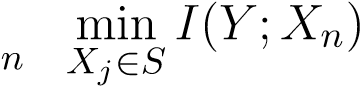
 *,* (1) *v*(*k*) = argmax
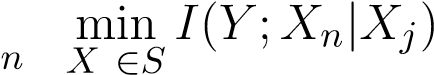
 *,* (2)

*j*

where *v* returns the index of the next selected feature. The Interaction Capping (ICAP) Jakulin (2005) criterion adds a min penalty term for feature redundancy

*J_ICAP_*(*X_i_*) = *I*(*X_i_*;*C*) + ^X^ min(0*,I*(*X_i_*;*s_j_*;*C*))*.* (3)

*s_j_*∈*S*

Joint Mutual Information (JMI) Bennasar et al. (2015) is a generalization of MI for a number of variables: *I*(*X_i_,...,X_k_*;*Y* ) = *KL*(*p*(*x_i_,...,x_k_,y*)||*p*(*x_i_,...,x_k_*)*p*(*y*))*.*

In the original work, it has been used to select inputs for a neural network, and applied with joint pdfs for pairs of variables. In FEAST, it is also applied to pairs, where the final score is computed as

*Jjmi* = X *I*(*Xk,Xj*;*Y* )*.*

*X_j_*∈*S*

Feature selection with Minimum redundancy maximum relevance (mRMR) Peng et al. (2005) is based on mutual information. It maximizes relevance given as

*D*(*S,c*) = 1*/*|*S*| ^X^ *I*(*x_i_*;*c*)*,*

*x_i_*∈*S*

where *I*(*x*;*c*) denotes mutual information, *S* feature set and *c* a particular class, while minimizing redundancy

*R*(*S*) = 1*/*|*S*|^2 X^ *I*(*x_i_*;*x_j_*)*.*

*x_i_,x_j_*∈*S*

As the method works best with categorical data, expression levels have been quantized into 10 equallyspaced bins. Score of the *i*th feature is computed as

*SNR*
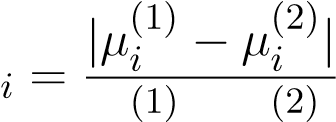
*,*

*σi* + *σi*

where
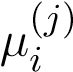
 denotes mean and
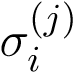
 variance of class *j*. Higher values of SNR signify stronger bond with one of the classes. The attributes are sorted in the descending order of their Signal-to-Noise Ratio (SNRs). It is applied greedily over all features, where top *k* are returned. The test can be applied as a simple method of feature selection, when it works on the data expressed through ranks Troyanskaya et al. (2002). As a feature selection method, it tests equality of data restricted to each of the features alone. For each of the classes, it tests the mean against all other background classes. The other feature selection methods such as Conditional Infomax Feature Extraction (CIFE)and Double Input Symmetrical Relevance (DISR) can be found in Brown et al. (2012b).

# RESULTS

**Table S1.** List of 39 miRNAs with their classification accuracy using Random Forest classification

| Serial Number | miRNA | Accuracy | Serial Number | miRNA | Accuracy | Serial Number | miRNA | Accuracy | Serial Number | miRNA | Accuracy |
| --- | --- | --- | --- | --- | --- | --- | --- | --- | --- | --- | --- |
| 1 | hsa-mir-205 | 74*.*21±0*.*0384 | 11 | hsa-mir-1 | 76*.*88±0*.*0404 | 21 | hsa-mir-125a |  | 31 | hsa-mir-577 |  |
| 2 | hsa-mir-10a |  | 12 | hsa-mir-30c | 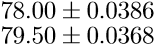 | 22 | hsa-mir-152 |  | 32 | hsa-let-7f |  |
| 3 | hsa-mir-196b | 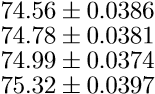 | 13 | hsa-mir-16 |  | 23 | hsa-mir-101 | 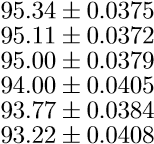 | 33 | hsa-mir-99a |  |
| 4 | hsa-mir-10b |  | 14 | hsa-mir-30a |  | 24 | hsa-mir-148a |  | 34 | hsa-mir-145 |  |
| 5 | hsa-mir-375 |  | 15 | hsa-let-7i |  | 25 | hsa-mir-184 |  | 35 | hsa-mir-149 | 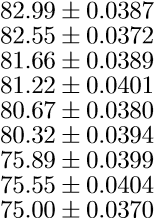 |
| 6 | hsa-mir-143 |  | 16 | hsa-mir-24 |  | 26 | hsa-mir-194 |  | 36 | hsa-mir-326 |  |
| 7 | hsa-let-7c |  | 17 | hsa-mir-95 | 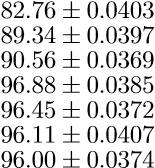 | 27 | hsa-mir-628 |  | 37 | hsa-mir-362 |  |
| 8 | hsa-mir-107 | 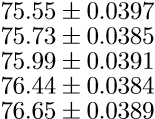 | 18 | hsa-mir-27b |  | 28 | hsa-mir-28 | 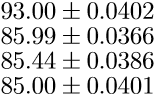 | 38 | hsa-mir-455 |  |
| 9 | hsa-mir-378 |  | 19 | hsa-mir-135b |  | 29 | hsa-mir-139 |  | 39 | hsa-mir-193a |  |
| 10 | hsa-mir-133a |  | 20 | hsa-mir-584 |  | 30 | hsa-mir-125b |  |  |  |  |

**Table S2.** Regulation and FDR of each selected miRNA for ten cancer types

| miRNA | BLCA Regulation FDR | | BRCA Regulation FDR | | COAD Regulation FDR | | GBM  Regulation FDR | | HNSC Regulation FDR | | KIRC Regulation FDR | | LUAD Regulation FDR | | LUSC Regulation FDR | | OV  Regulation | FDR | UCEC Regulation FDR | |
| --- | --- | --- | --- | --- | --- | --- | --- | --- | --- | --- | --- | --- | --- | --- | --- | --- | --- | --- | --- | --- |
|  | (up↑/down↓) |  | (up↑/down↓) |  | (up↑/down↓) |  | (up↑/down↓) |  | (up↑/down↓) |  | (up↑/down↓) |  | (up↑/down↓) | | (up↑/down↓) |  | (up↑/down↓) |  | (up↑/down↓) | |
| hsa-mir-205 | ↑ | 1.74E-15 | ↑ | 1.44E-06 | ↓ | 3.29E-27 | ↓ | 5.46E-03 | ↑ | 2.23E-56 | ↓ | 2.88E-48 | - | 4.82E-01 | ↑ | 1.16E-24 | ↑ | 2.08E-03 | - | 1.05E-01 |
| hsa-mir-10a | ↓ | 1.01E-11 | ↓ | 2.46E-09 | ↑ | 2.77E-19 | ↓ | 1.87E-23 | ↓ | 6.42E-72 | ↓ | 1.44E-05 | ↓ | 1.57E-04 | - | 9.34E-01 | ↓ | 6.35E-121 | ↑ | 1.68E-20 |
| hsa-mir-196b | ↑ | 1.29E-09 | ↑ | 4.42E-10 | ↑ | 2.92E-57 | - | 8.62E-01 | ↑ | 2.50E-02 | ↑ | 1.82E-34 | - | 3.03E-01 | ↑ | 5.75E-12 | ↓ | 1.42E-03 | ↑ | 1.21E-47 |
| hsa-mir-10b | ↓ | 5.28E-07 | ↓ | 4.35E-04 | ↓ | 2.05E-05 | ↓ | 5.39E-21 | ↓ | 1.48E-18 | ↑ | 1.72E-02 | ↓ | 6.00E-17 | ↓ | 4.08E-12 | ↓ | 2.27E-116 | ↓ | 4.71E-03 |
| hsa-mir-375 | ↓ | 3.90E-14 | ↑ | 6.40E-50 | ↑ | 4.59E-46 | ↓ | 2.36E-14 | ↓ | 3.96E-14 | ↓ | 4.87E-42 | ↑ | 2.02E-21 | - | 4.24E-01 | ↓ | 2.00E-98 | ↑ | 1.25E-11 |
| hsa-mir-143 | ↑ | 8.32E-05 | ↓ | 1.32E-65 | ↓ | 1.82E-02 | ↓ | 1.87E-23 | ↓ | 1.14E-04 | ↓ | 3.78E-16 | ↓ | 6.28E-09 | ↓ | 4.98E-09 | ↓ | 1.79E-56 | ↓ | 1.07E-27 |
| hsa-let-7c | ↓ | 1.31E-37 | ↓ | 3.89E-37 | ↓ | 5.88E-58 | ↑ | 6.32E-16 | ↓ | 1.83E-45 | ↓ | 7.84E-45 | ↓ | 2.84E-31 | ↓ | 2.53E-27 | ↓ | 5.14E-29 | ↓ | 4.57E-27 |
| hsa-mir-107 | ↑ | 7.95E-49 | ↑ | 2.71E-95 | ↓ | 7.99E-03 | ↑ | 1.87E-23 | ↑ | 1.18E-103 | ↑ | 2.69E-67 | ↑ | 1.14E-34 | ↑ | 7.17E-47 | ↑ | 1.13E-131 | ↑ | 1.84E-50 |
| hsa-mir-378 | ↓ | 3.19E-17 | ↓ | 8.75E-79 | ↑ | 2.89E-06 | ↓ | 7.79E-20 | ↓ | 8.63E-29 | ↓ | 2.23E-42 | ↓ | 2.01E-31 | ↓ | 9.98E-34 | ↓ | 1.04E-64 | ↓ | 6.39E-39 |
| hsa-mir-133a | ↑ | 1.21E-22 | ↑ | 1.35E-22 | ↑ | 1.93E-36 | ↑ | 4.73E-20 | ↑ | 4.05E-54 | ↑ | 5.08E-25 | ↑ | 1.71E-13 | ↑ | 3.75E-14 | ↑ | 5.13E-99 | ↑ | 5.81E-22 |
| hsa-mir-1 | ↓ | 1.38E-03 | ↓ | 3.26E-60 | - | 5.45E-01 | ↑ | 1.04E-07 | - | 8.11E-01 | ↓ | 3.74E-27 | ↓ | 1.22E-07 | ↓ | 1.77E-15 | - | 1.84E-01 | ↓ | 1.34E-11 |
| hsa-mir-30c | ↓ | 3.16E-35 | - | 1.61E-01 | ↓ | 6.34E-17 | ↓ | 2.93E-13 | ↓ | 1.39E-80 | ↑ | 4.82E-08 | ↓ | 1.12E-14 | ↓ | 8.70E-30 | ↓ | 4.02E-04 | - | 1.72E-01 |
| hsa-mir-16 | ↑ | 7.95E-49 | ↑ | 2.71E-95 | ↑ | 4.31E-58 | ↑ | 1.87E-23 | ↑ | 1.18E-103 | ↑ | 2.69E-67 | ↑ | 1.14E-34 | ↑ | 7.17E-47 | ↑ | 1.79E-132 | ↑ | 1.84E-50 |
| hsa-mir-30a | ↓ | 2.62E-44 | ↓ | 2.21E-06 | ↓ | 9.80E-56 | ↓ | 2.14E-23 | ↓ | 1.09E-95 | ↑ | 6.97E-18 | ↓ | 4.41E-17 | ↓ | 9.12E-34 | ↓ | 1.79E-132 | ↓ | 2.52E-17 |
| hsa-let-7i | ↓ | 2.37E-12 | ↑ | 2.83E-43 | ↓ | 6.17E-25 | ↑ | 2.69E-21 | ↑ | 6.45E-07 | ↑ | 3.02E-38 | ↑ | 4.98E-12 | ↑ | 8.27E-08 | ↑ | 3.25E-125 | - | 4.71E-01 |
| hsa-mir-24 | ↓ | 7.95E-49 | ↓ | 2.71E-95 | ↓ | 4.31E-58 | ↓ | 1.87E-23 | ↓ | 1.18E-103 | ↓ | 2.69E-67 | ↓ | 1.14E-34 | ↓ | 7.17E-47 | ↓ | 1.79E-132 | ↓ | 1.84E-50 |
| hsa-mir-95 | ↓ | 8.18E-07 | ↑ | 3.91E-11 | ↑ | 3.60E-44 | ↑ | 1.87E-23 | ↑ | 4.87E-22 | ↑ | 2.77E-09 | ↑ | 3.27E-04 | - | 3.79E-01 | ↑ | 2.75E-125 | ↑ | 1.39E-13 |


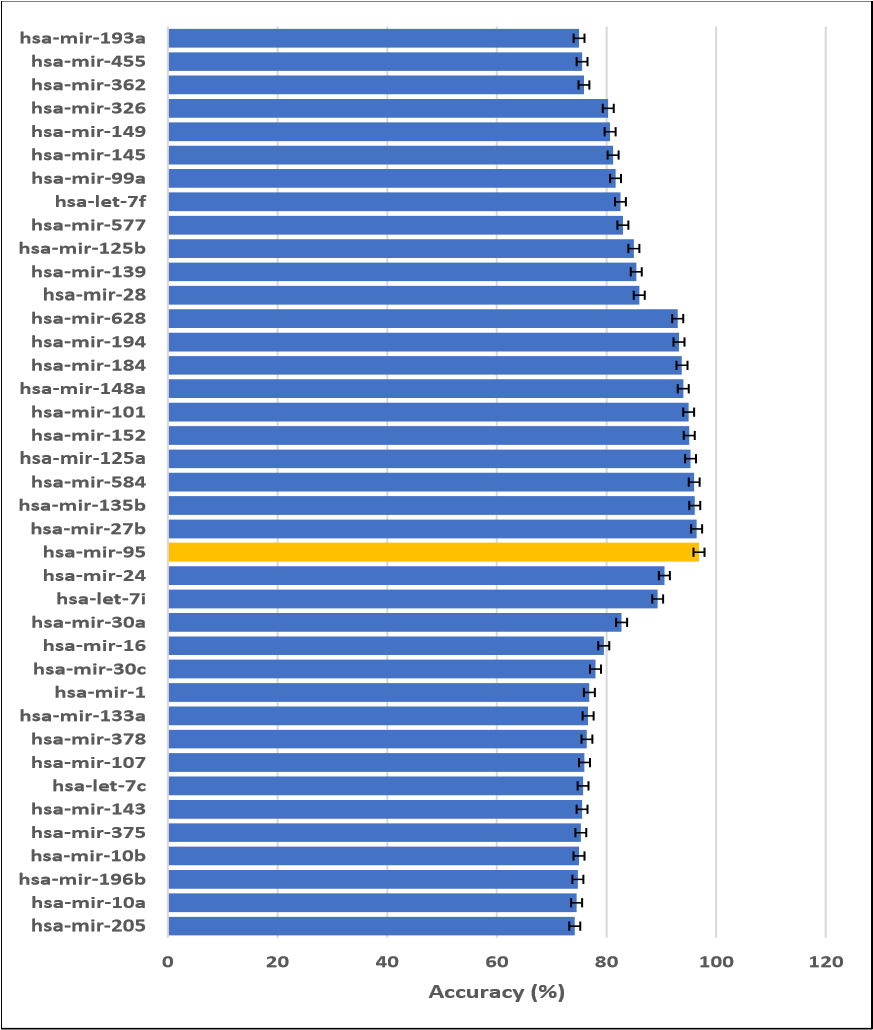


**Figure S1.** Barchart representation of classification accuracy (RF) of miRNAs for performing multi-class classification of ten diverse cancer types. The higher accuracy is archived by considering 39 miRNAs and that has been marked with yellow color

86

88

90

92

94

96

98

100

0

20

40

60

80

100

10

20

30

40

50

60

70

80

90

Accuracy [%]

#Features

Generation

Accuracy

Number of features

86

88

90

92

94

96

98

100

0

20

40

60

80

100

10

20

30

40

50

60

70

80

90

Accuracy [%]

#Features

Generation

Accuracy

Number of features

(a) (b)

86

88

90

92

94

96

98

100

0

20

40

60

80

100

10

20

30

40

50

60

70

80

Accuracy [%]

#Features

Generation

Accuracy

Number of features

86

88

90

92

94

96

98

100

0

20

40

60

80

100

10

20

30

40

50

60

70

80

90

Accuracy [%]

#Features

Generation

Accuracy

Number of features

(c) (d)

**Figure S2.** Performance of the proposed SCES feature selection method with (a) RF, (b) SVM, (c) NB and(d) K-NN classifiers and representation based on SNE. The figures depict single SCES runs. Classification accuracy varies during rapid decrease of the number of features, and converges near the end after nearly 90 generations


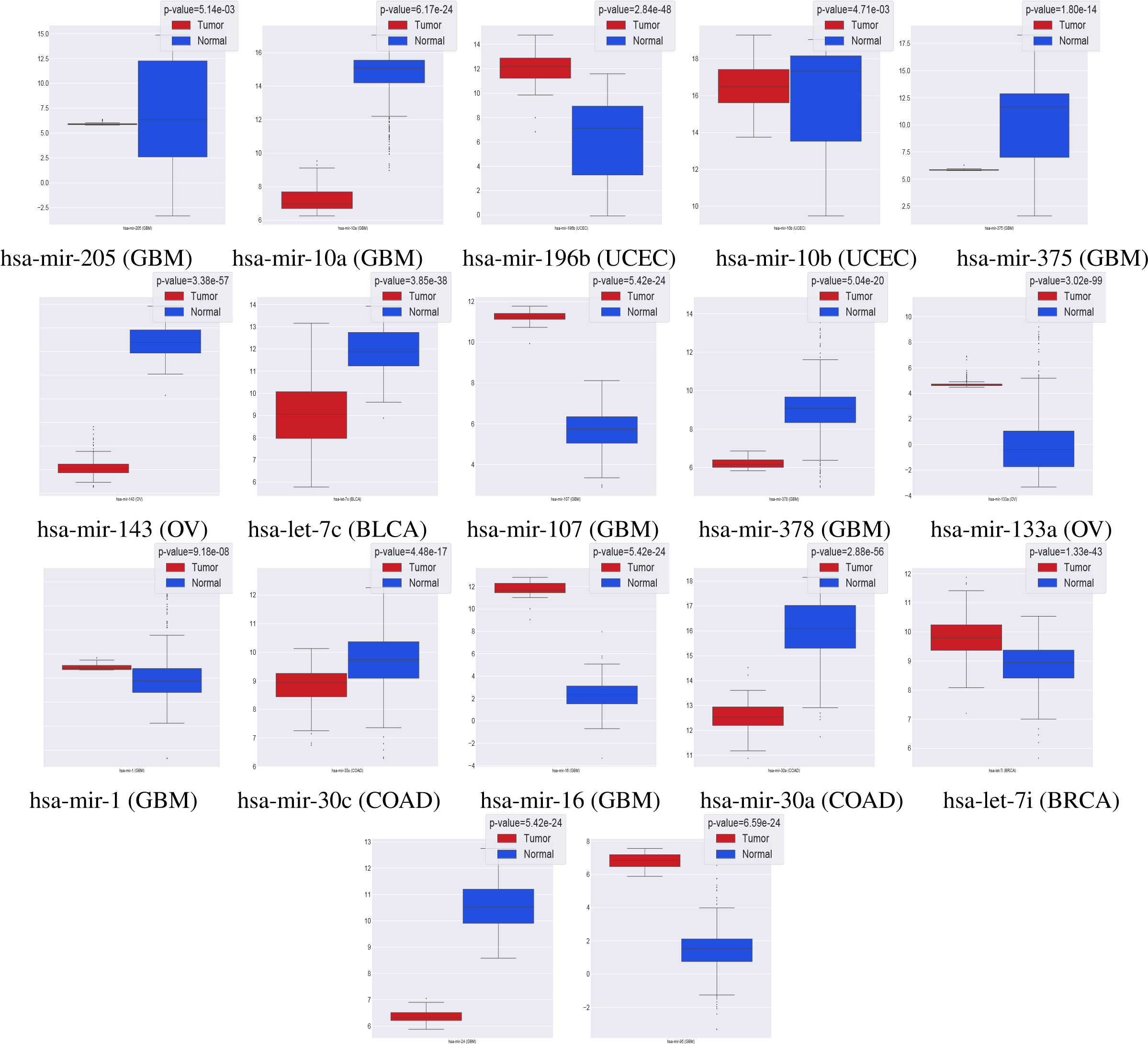


hsa-mir-24 (GBM) hsa-mir-95 (GBM)

**Figure S3**. Expression analysis using box plots of expression values for hsa-mir-205 (GBM), hsa-mir-10a (GBM), hsa-mir-196b (UCEC), hsa-mir-10b (UCEC), hsa-mir-375 (GBM), hsa-mir-143 (OV), hsa-let-7c (BLCA), hsa-mir-107 (GBM), hsa-mir-378 (GBM), hsa-mir-133a (OV), hsa-mir-1 (GBM), hsa-mir-30c (COAD), hsa-mir-16 (GBM), hsa-mir-30a (COAD), hsa-let-7i (BRCA), hsa-mir-24 (GBM) and hsa-mir-95(GBM)





**Figure S4.** full-length of figure 4 gels are included in this Supplementary Information

**Table S3** Association of selected 17 miRNAs and their targets in ten diverse cancer types

| miRNA-targets |  |  | Association in different cancer types | | | | |  |  |  | Association  Count | Cumulative  Corr. Score |
| --- | --- | --- | --- | --- | --- | --- | --- | --- | --- | --- | --- | --- |
|  | BLCA | BRCA | COAD | GBM | HNSC | KIRC | LUAD | LUSC | OV | UCEC |  |  |
| hsa-mir-205-CYR61 | 3 | - | 3 | - | - | - | - | 3 | - | - | 3 | -1.716 |
| hsa-mir-205-CTGF | 3 | - | 3 | - | - | - | - | 3 | - | - | 3 | -1.653 |
| hsa-mir-205-TNFSF8 | 3 | - | 3 | - | - | - | - | 3 | - | - | 3 | -1.621 |
| hsa-mir-205-SLC7A2 | - | - | 3 | - | - | - | - | - | - | 3 | 2 | -1.517 |
| hsa-mir-205-PDLIM5 | - | - | 3 | 3 | - | - | - | - | - | - | 2 | -1.379 |
| hsa-mir-205-STARD8 | 3 | - | - | - | - | - | - | 3 | - | - | 2 | -1.311 |
| hsa-mir-205-TIMP1 | - | - | 3 | - | - | - | - | - | - | - | 1 | -0.963 |
| hsa-mir-205-SESN3 | - | - | 3 | - | - | - | - | - | - | - | 1 | -0.943 |
| hsa-mir-205-SAMD8 | - | - | 3 | - | - | - | - | - | - | - | 1 | -0.899 |
| hsa-mir-10a-TTYH3 | - | - | 3 | - | - | 3 | - | - | - | - | 2 | -1.261 |
| hsa-mir-10a-LILRA2 | - | - | 3 | - | - | 3 | - | - | - | - | 2 | -1.220 |
| hsa-mir-10a-CARHSP1 | - | - | 3 | - | - | 3 | - | - | - | - | 2 | -1.197 |
| hsa-mir-10a-CD3D | - | - | 3 | - | - | 3 | - | - | - | - | 2 | -1.176 |
| hsa-mir-10a-H3F3C | - | - | 3 | - | - | - | - | - | - | - | 1 | -0.962 |
| hsa-mir-10a-FHL2 | - | - | 3 | - | - | - | - | - | - | - | 1 | -0.911 |
| hsa-mir-10a-MTR | - | - | 3 | - | - | - | - | - | - | - | 1 | -0.899 |
| hsa-mir-10a-SFT2D2 | - | - | 3 | - | - | - | - | - | - | - | 1 | -0.882 |
| hsa-mir-196b-GATA6 | 3 | - | 3 | - | - | - | - | 3 | - | - | 3 | -1.877 |
| hsa-mir-196b-LAMB2 | - | - | 3 | - | - | - | - | 3 | - | - | 2 | -1.139 |
| hsa-mir-196b-MAP2K2 | - | - | 3 | 3 | - | - | - | - | - | - | 2 | -1.124 |
| hsa-mir-196b-KCTD21 | - | - | 3 | - | - | - | - | - | - | - | 1 | -0.989 |
| hsa-mir-196b-ACER2 | - | - | 3 | - | - | - | - | - | - | - | 1 | -0.974 |
| hsa-mir-196b-BCAR3 | - | - | 3 | - | - | - | - | - | - | - | 1 | -0.968 |
| hsa-mir-196b-IGF2BP3 | - | - | 3 | - | - | - | - | - | - | - | 1 | -0.943 |
| hsa-mir-196b-HIST1H2BD | - | - | 3 | - | - | - | - | - | - | - | 1 | -0.910 |
| hsa-mir-196b-CDK5R1 | - | - | 3 | - | - | - | - | - | - | - | 1 | -0.903 |
| hsa-mir-10b-POC1A | - | 3 | 3 | - | - | - | - | - | - | - | 2 | -1.392 |
|  | |  |  | | | | |  |  | Continued on next page | | |

| miRNA-mRNA |  |  | Association in 10 diverse cancer types | | | | |  |  |  | Association  Count | Cumulative  Corr. Score |
| --- | --- | --- | --- | --- | --- | --- | --- | --- | --- | --- | --- | --- |
|  | BLCA | BRCA | COAD | GBM | HNSC | KIRC | LUAD | LUSC | OV | UCEC |  |  |
| hsa-mir-10b-UHRF1 | - | 3 | 3 | - | - | - | - | - | - | - | 2 | -1.236 |
| hsa-mir-10b-TUBA1B | - | 3 | - | - | - | 3 | - | - | - | - | 2 | -1.106 |
| hsa-mir-10b-INHBA | - | - | 3 | - | - | - | - | - | - | - | 1 | -0.991 |
| hsa-mir-10b-MBNL3 | - | - | 3 | - | - | - | - | - | - | - | 1 | -0.982 |
| hsa-mir-10b-OPA3 | - | - | 3 | - | - | - | - | - | - | - | 1 | -0.961 |
| hsa-mir-10b-SLC2A3 | - | - | 3 | - | - | - | - | - | - | - | 1 | -0.955 |
| hsa-mir-10b-PPP1R13B | - | - | 3 | - | - | - | - | - | - | - | 1 | -0.935 |
| hsa-mir-10b-CD3D | - | - | 3 | - | - | - | - | - | - | - | 1 | -0.928 |
| hsa-mir-375-CRIM1 | - | - | 3 | - | - | - | 3 | - | - | - | 2 | -1.306 |
| hsa-mir-375-CFL2 | - | 3 | 3 | - | - | - | - | - | - | - | 2 | -1.258 |
| hsa-mir-375-RHOQ | - | 3 | 3 | - | - | - | - | - | - | - | 2 | -1.258 |
| hsa-mir-375-TNS1 | - | 3 | 3 | - | - | - | - | - | - | - | 2 | -1.204 |
| hsa-mir-375-SON | - | - | 3 | - | - | - | - | - | - | - | 1 | -0.994 |
| hsa-mir-375-LIMD2 | - | - | 3 | - | - | - | - | - | - | - | 1 | -0.981 |
| hsa-mir-375-ATG7 | - | - | 3 | - | - | - | - | - | - | - | 1 | -0.980 |
| hsa-mir-375-JAK2 | - | - | 3 | - | - | - | - | - | - | - | 1 | -0.958 |
| hsa-mir-143-RAB10 | - | - | 3 | - | - | - | 3 | - | - | - | 2 | -1.264 |
| hsa-mir-143-CENPM | - | 3 | 3 | - | - | - | - | - | - | - | 2 | -1.144 |
| hsa-mir-143-NKPD1 | - | - | 3 | - | - | - | - | - | - | - | 1 | -0.987 |
| hsa-mir-143-TIAL1 | - | - | 3 | - | - | - | - | - | - | - | 1 | -0.981 |
| hsa-mir-143-TUBD1 | - | - | 3 | - | - | - | - | - | - | - | 1 | -0.951 |
| hsa-mir-143-PHAX | - | - | 3 | - | - | - | - | - | - | - | 1 | -0.948 |
| hsa-mir-143-TTC38 | - | - | 3 | - | - | - | - | - | - | - | 1 | -0.944 |
| hsa-mir-143-FADS6 | - | - | 3 | - | - | - | - | - | - | - | 1 | -0.936 |
| hsa-let-7c-LDHA | - | - | 3 | - | 3 | - | 3 | - | - | - | 3 | -1.951 |
| hsa-let-7c-MRPL12 | - | 3 | 3 | - | - | - | 3 | - | - | - | 3 | -1.883 |
| hsa-let-7c-BRI3BP | - | 3 | 3 | - | - | - | 3 | - | - | - | 3 | -1.863 |
| hsa-let-7c-YWHAZ | - | - | 3 | - | - | - | 3 | - | - | - | 2 | -1.643 |
| hsa-let-7c-E2F5 | - | - | 3 | - | - | - | 3 | - | - | - | 2 | -1.466 |
|  | |  |  | | | | |  |  | Continued on next page | | |

| miRNA-mRNA |  |  | Association in 10 diverse cancer types | | | | |  |  |  | Association  Count | Cumulative  Corr. Score |
| --- | --- | --- | --- | --- | --- | --- | --- | --- | --- | --- | --- | --- |
|  | BLCA | BRCA | COAD | GBM | HNSC | KIRC | LUAD | LUSC | OV | UCEC |  |  |
| hsa-let-7c-PTMA | - | - | 3 | - | - | - | 3 | - | - | - | 2 | -1.356 |
| hsa-let-7c-HMGXB4 | - | - | 3 | - | - | - | - | - | - | - | 1 | -0.981 |
| hsa-let-7c-HES5 | - | - | 3 | - | - | - | - | - | - | - | 1 | -0.980 |
| hsa-let-7c-WDR3 | - | - | 3 | - | - | - | - | - | - | - | 1 | -0.967 |
| hsa-mir-107-CPEB3 | 3 | - | - | - | 3 | 3 | 3 | 3 | - | - | 5 | -3.224 |
| hsa-mir-107-TGFBR3 | 3 | 3 | - | - | - | 3 | 3 | - | - | - | 4 | -2.958 |
| hsa-mir-107-FGF2 | 3 | 3 | 3 | - | - | - | 3 | - | - | - | 4 | -2.861 |
| hsa-mir-107-CAV1 | - | 3 | - | - | - | - | 3 | 3 | - | - | 3 | -2.285 |
| hsa-mir-107-KLF4 | 3 | 3 | - | - | - | - | 3 | - | - | - | 3 | -2.089 |
| hsa-mir-107-RS1 | - | - | - | - | - | - | 3 | 3 | - | - | 2 | -1.763 |
| hsa-mir-107-SH3GL2 | - | - | - | - | - | 3 | 3 | - | - | - | 2 | -1.695 |
| hsa-mir-107-TMEM87A | - | - | 3 | - | - | - | - | - | - | - | 1 | -0.963 |
| hsa-mir-107-CDC42SE2 | - | - | 3 | - | - | - | - | - | - | - | 1 | -0.952 |
| hsa-mir-378-SERPINH1 | - | - | 3 | - | 3 | - | 3 | - | - | - | 3 | -1.907 |
| hsa-mir-378-HIST1H2BD | - | 3 | 3 | - | - | - | 3 | - | - | - | 3 | -1.828 |
| hsa-mir-378-NWD1 | - | - | 3 | 3 | - | - | - | - | - | - | 2 | -1.552 |
| hsa-mir-378-NME4 | - | - | 3 | - | - | - | 3 | - | - | - | 2 | -1.505 |
| hsa-mir-378-ORAI2 | - | 3 | 3 | - | - | - | - | - | - | - | 2 | -1.480 |
| hsa-mir-378-TMEM154 | - | - | 3 | - | - | - | - | - | - | - | 1 | -0.976 |
| hsa-mir-378-MYADM | - | - | 3 | - | - | - | - | - | - | - | 1 | -0.969 |
| hsa-mir-378-OPA3 | - | - | 3 | - | - | - | - | - | - | - | 1 | -0.957 |
| hsa-mir-133a-TMEM59 | - | - | 3 | - | - | 3 | - | - | - | - | 2 | -1.523 |
| hsa-mir-133a-KRT7 | - | - | 3 | - | - | 3 | - | - | - | - | 2 | -1.388 |
| hsa-mir-133a-TCTEX1D2 | - | - | 3 | - | - | 3 | - | - | - | - | 2 | -1.280 |
| hsa-mir-133a-PNP | - | - | 3 | - | - | 3 | - | - | - | - | 2 | -1.192 |
| hsa-mir-133a-UGT2B10 | - | - | 3 | - | - | - | - | - | - | - | 1 | -0.930 |
| hsa-mir-133a-CDC42 | - | - | 3 | - | - | - | - | - | - | - | 1 | -0.908 |
| hsa-mir-133a-SEC61B | - | - | 3 | - | - | - | - | - | - | - | 1 | -0.903 |
| hsa-mir-133a-MYL12A | - | - | 3 | - | - | - | - | - | - | - | 1 | -0.803 |
|  | |  |  | | | | |  |  | Continued on next page | | |

| miRNA-mRNA |  |  | Association in 10 diverse cancer types | | | | |  |  |  | Association  Count | Cumulative  Corr. Score |
| --- | --- | --- | --- | --- | --- | --- | --- | --- | --- | --- | --- | --- |
|  | BLCA | BRCA | COAD | GBM | HNSC | KIRC | LUAD | LUSC | OV | UCEC |  |  |
| hsa-mir-1-SRP19 | 3 | - | 3 | - | - | - | - | - | - | - | 2 | -1.515 |
| hsa-mir-1-GNG5 | 3 | - | 3 | - | - | - | - | - | - | - | 2 | -1.456 |
| hsa-mir-1-CAPZA1 | 3 | - | 3 | - | - | - | - | - | - | - | 2 | -1.402 |
| hsa-mir-1-DPY30 | 3 | - | 3 | - | - | - | - | - | - | - | 2 | -1.373 |
| hsa-mir-1-BMP7 | - | - | 3 | - | - | - | - | - | - | - | 1 | -0.988 |
| hsa-mir-1-CAST | - | - | 3 | - | - | - | - | - | - | - | 1 | -0.973 |
| hsa-mir-1-CEBPA | - | - | 3 | - | - | - | - | - | - | - | 1 | -0.972 |
| hsa-mir-1-GOLGA7 | - | - | 3 | - | - | - | - | - | - | - | 1 | -0.967 |
| hsa-mir-30c-UBE2I | - | - | 3 | - | - | 3 | - | - | - | - | 2 | -1.456 |
| hsa-mir-30c-VIM | - | - | 3 | - | - | 3 | - | - | - | - | 2 | -1.349 |
| hsa-mir-30c-SERPINE1 | - | - | 3 | - | - | 3 | - | - | - | - | 2 | -1.327 |
| hsa-mir-30c-LHFPL2 | - | - | 3 | - | - | 3 | - | - | - | - | 2 | -1.301 |
| hsa-mir-30c-SOCS3 | - | - | 3 | - | - | - | - | - | - | - | 1 | -0.978 |
| hsa-mir-30c-ARF3 | - | - | 3 | - | - | - | - | - | - | - | 1 | -0.949 |
| hsa-mir-30c-FOXA1 | - | - | 3 | - | - | - | - | - | - | - | 1 | -0.948 |
| hsa-mir-30c-CADPS2 | - | - | 3 | - | - | - | - | - | - | - | 1 | -0.843 |
| hsa-mir-16-PHYHIP | 3 | 3 | 3 | 3 | - | 3 | - | - | - | 3 | 6 | -4.142 |
| hsa-mir-16-CPEB3 | 3 | 3 | - | - | 3 | 3 | 3 | 3 | - | - | 6 | -3.825 |
| hsa-mir-16-DIXDC1 | 3 | 3 | 3 | - | - | - | 3 | 3 | - | - | 5 | -3.715 |
| hsa-mir-16-RUNX1T1 | 3 | 3 | 3 | - | - | - | 3 | 3 | - | - | 5 | -3.642 |
| hsa-mir-16-SLC6A4 | - | - | - | - | - | 3 | 3 | 3 | - | 3 | 4 | -3.172 |
| hsa-mir-16-CTDSPL | - | - | 3 | - | - | 3 | 3 | 3 | - | - | 4 | -3.003 |
| hsa-mir-16-RS1 | - | - | 3 | - | - | - | 3 | 3 | - | - | 3 | -2.485 |
| hsa-mir-16-OSCAR | - | - | 3 | - | - | - | 3 | 3 | - | - | 3 | -2.393 |
| hsa-mir-16-STK33 | - | - | 3 | - | - | 3 | - | - | - | - | 2 | -1.697 |
| hsa-mir-16-FLNA | 3 | - | 3 | - | - | - | - | - | - | - | 2 | -1.660 |
| hsa-mir-16-CLIP2 | - | - | 3 | - | - | - | - | - | - | - | 1 | -0.996 |
| hsa-mir-16-CAMSAP1 | - | - | 3 | - | - | - | - | - | - | - | 1 | -0.995 |
| hsa-mir-30a-CDC20 | - | - | 3 | - | - | 3 | 3 | 3 | - | - | 4 | -2.470 |
|  | |  |  | | | | |  |  | Continued on next page | | |

| miRNA-mRNA |  |  | Association in 10 diverse cancer types | | | | |  |  |  | Association  Count | Cumulative  Corr. Score |
| --- | --- | --- | --- | --- | --- | --- | --- | --- | --- | --- | --- | --- |
|  | BLCA | BRCA | COAD | GBM | HNSC | KIRC | LUAD | LUSC | OV | UCEC |  |  |
| hsa-mir-30a-MYBL2 | - | - | 3 | - | - | 3 | 3 | 3 | - | - | 4 | -2.332 |
| hsa-mir-30a-SFXN1 | - | - | 3 | - | - | - | 3 | 3 | - | - | 3 | -2.089 |
| hsa-mir-30a-MTHFD2 | - | - | 3 | - | - | 3 | 3 | - | - | - | 3 | -1.772 |
| hsa-mir-30a-SOX12 | - | - | 3 | - | - | - | 3 | - | - | - | 2 | -1.439 |
| hsa-mir-30a-PES1 | - | - | 3 | 3 | - | - | - | - | - | - | 2 | -1.389 |
| hsa-mir-30a-NCEH1 | - | - | 3 | - | - | - | - | - | - | - | 1 | -0.964 |
| hsa-mir-30a-SBF1 | - | - | 3 | - | - | - | - | - | - | - | 1 | -0.961 |
| hsa-let-7i-TUBB2A | - | - | 3 | - | - | 3 | - | - | - | - | 2 | -1.567 |
| hsa-let-7i-MSI2 | - | - | 3 | - | - | 3 | - | - | - | - | 2 | -1.491 |
| hsa-let-7i-RAB11FIP4 | - | - | 3 | - | - | 3 | - | - | - | - | 2 | -1.383 |
| hsa-let-7i-PRSS22 | - | - | 3 | - | - | 3 | - | - | - | - | 2 | -1.306 |
| hsa-let-7i-SLC20A1 | - | - | 3 | - | - | - | - | - | - | - | 1 | -0.929 |
| hsa-let-7i-SURF4 | - | - | 3 | - | - | - | - | - | - | - | 1 | -0.886 |
| hsa-let-7i-ACOT9 | - | - | 3 | - | - | - | - | - | - | - | 1 | -0.867 |
| hsa-let-7i-ZNF200 | - | - | 3 | - | - | - | - | - | - | - | 1 | -0.838 |
| hsa-mir-24-C1QTNF6 | 3 | 3 | - | - | 3 | 3 | 3 | 3 | - | - | 6 | -3.906 |
| hsa-mir-24-PKMYT1 | 3 | 3 | - | - | 3 | 3 | 3 | 3 | - | - | 6 | -3.873 |
| hsa-mir-24-UBE2C | 3 | 3 | - | - | - | 3 | 3 | 3 | - | - | 5 | -3.500 |
| hsa-mir-24-RRM2 | 3 | 3 | - | - | - | 3 | 3 | 3 | - | - | 5 | -3.357 |
| hsa-mir-24-CCNB1 | 3 | 3 | - | - | - | - | 3 | 3 | - | - | 4 | -2.825 |
| hsa-mir-24-CDK1 | 3 | 3 | - | - | - | - | 3 | 3 | - | - | 4 | -2.780 |
| hsa-mir-24-NEK6 | - | - | 3 | - | - | 3 | 3 | - | - | - | 3 | -1.995 |
| hsa-mir-24-ALDOA | - | 3 | - | - | - | 3 | 3 | - | - | - | 3 | -1.995 |
| hsa-mir-24-IGSF6 | - | - | 3 | - | - | 3 | - | - | - | - | 2 | -1.693 |
| hsa-mir-24-CLEC7A | - | - | 3 | - | - | 3 | - | - | - | - | 2 | -1.672 |
| hsa-mir-24-TTLL7 | - | - | 3 | - | - | - | - | - | - | - | 1 | -0.987 |
| hsa-mir-24-DCAF4 | - | - | 3 | - | - | - | - | - | - | - | 1 | -0.979 |
| hsa-mir-95-TBX18 | - | - | 3 | - | - | - | - | - | - | - | 1 | -0.974 |
| hsa-mir-95-MRAS | - | - | 3 | - | - | - | - | - | - | - | 1 | -0.852 |
|  | |  |  | | | | |  |  | Continued on next page | | |

| miRNA-mRNA |  |  | Association in 10 diverse cancer types |  |  |  | Association  Count | Cumulative  Corr. Score |
| --- | --- | --- | --- | --- | --- | --- | --- | --- |
|  | BLCA | BRCA | COAD GBM HNSC KIRC LUAD | LUSC | OV | UCEC |  |  |
| hsa-mir-95-ACVR1 | - | - | 3 - - - - | - | - | - | 1 | -0.839 |
| hsa-mir-95-WARS | - | - | 3 - - - - | - | - | - | 1 | -0.815 |
| hsa-mir-95-ARPC1B | - | - | 3 - - - - | - | - | - | 1 | -0.810 |
| hsa-mir-95-ARHGDIA | - | - | 3 - - - - | - | - | - | 1 | -0.804 |
| hsa-mir-95-SYT11 | - | - | 3 - - - - | - | - | - | 1 | -0.787 |
| hsa-mir-95-P2RY1 | - | - | 3 - - - - | - | - | - | 1 | -0.769 |
| hsa-mir-95-TMEM200C | - | - | 3 - - - - | - | - | - | 1 | -0.707 |
|  |  |  |  |  |  |  |  |  |
|  |  |  |  |  |  |  |  |  |

**Table S4.** *Five* significant GO Molecular Function for each selected 17 miRNAs in ten diverse cancer types

| miRNA | GO-Molecular Function |  |  |  | FDR corrected *p-value* | | | |  |  |  |
| --- | --- | --- | --- | --- | --- | --- | --- | --- | --- | --- | --- |
|  |  | BLCA | BRCA | COAD | GBM | HNSC | KIRC | LUAD | LUSC | OV | UCEC |
|  | GO:0008134 transcription factor binding | - | 8.10E-03 | - | 8.31E-06 | - | 6.80E-06 | 4.70E-03 | - | 3.76E-05 | 3.40E-03 |
|  | GO:0044877 protein-containing complex binding | - | - | - | 4.40E-03 | - | - | 4.70E-03 | 2.20E-03 | - | 1.19E-02 |
| hsa-mir-205 | GO:0005161 platelet-derived growth factor receptor binding | - | 1.64E-02 | - | - | - | - | - | 1.10E-03 | - | 1.14E-02 |
|  | GO:0019899 enzyme binding | - | - | 3.01E-02 | - | - | - | 4.70E-03 | 3.30E-03 | - | - |
|  | GO:0061629 RNA polymerase II-specific DNA-binding transcription factor binding | - | 1.56E-02 | - | - | - | - | - | - | 7.00E-03 | - |
|  | GO:1901363 heterocyclic compound binding | - | 4.85E-02 | - | - | - | - | 2.00E-03 | 6.30E-03 | 1.10E-03 | - |
|  | GO:0097159 organic cyclic compound binding | - | 4.85E-02 | - | - | - | - | 2.00E-03 | 6.30E-03 | 1.10E-03 | - |
| hsa-mir-10a | GO:0003723 RNA binding | - | 4.85E-02 | - | - | - | - | - | 6.30E-03 | - | - |
|  | GO:0003676 nucleic acid binding | - | 4.85E-02 | - | - | - | - | - | - | 2.57E-02 | - |
|  | GO:0016881 acid-amino acid ligase activity | - | 4.85E-02 | - | - | - | - | - | - | - | - |
|  | GO:0019899 enzyme binding | 8.37E-07 | 5.80E-04 | 6.76E-07 | - | 3.51E-06 | 4.68E-05 | 8.22E-07 | 9.69E-09 | 8.42E-07 | 1.33E-05 |
|  | GO:0019900 kinase binding | 3.00E-06 | 5.80E-04 | - | - | 3.51E-06 | - | 9.86E-05 | 6.72E-08 | 6.74E-05 | 1.00E-04 |
| hsa-mir-196b | GO:0019901 protein kinase binding | 1.31E-05 | 1.70E-03 | - | - | 1.36E-05 | 5.56E-05 | 4.20E-04 | 2.00E-06 | - | - |
|  | GO:1990837 sequence-specific double-stranded DNA binding | - | - | 1.48E-06 | 2.90E-04 | - | 5.56E-05 | 4.20E-04 | - | - | 1.20E-03 |
|  | GO:0003690 double-stranded DNA binding | 1.10E-04 | - | 6.76E-07 | 1.10E-04 | - | - | - | - | - | 8.70E-04 |
|  | GO:0005488 binding | 5.20E-03 | - | - | 2.79E-02 | - | - | 3.40E-02 | - | - | 1.11E-02 |
|  | GO:0005515 protein binding | 5.20E-03 | - | - | 3.02E-02 | - | - | - | - | 8.00E-04 | - |
| hsa-mir-10b | GO:1901363 heterocyclic compound binding | - | 4.00E-04 | - | - | - | - | 3.40E-02 | - | - | - |
|  | GO:0097159 organic cyclic compound binding | - | 4.00E-04 | - | - | - | - | 3.40E-02 | - | - | - |
|  | GO:0001221 transcription cofactor binding | 7.80E-03 | - | - | - | - | - | - | - | - | - |
|  | GO:0005515 protein binding | 1.11E-02 | 1.20E-03 | 9.58E-05 | - | - | - | 3.08E-07 | - | 1.50E-03 | 3.05E-05 |
|  | GO:0005488 binding | - | 3.10E-03 | - | - | - | - | 2.40E-03 | - | - | 3.00E-03 |
| hsa-mir-375 | GO:0019904 protein domain specific binding | - | - | 1.49E-02 | - | - | - | 1.01E-02 | - | 5.60E-03 | - |
|  | GO:0043548 phosphatidylinositol 3-kinase binding | 8.00E-04 | 4.97E-02 | - | - | - | - | - | - | - | - |
|  | GO:0005010 insulin-like growth factor-activated receptor activity | 1.11E-02 | - | - | - | - | - | - | - | - | 1.98E-02 |
|  | GO:0048407 platelet-derived growth factor binding | - | - | - | - | - | - | 1.51E-02 | - | - | - |
|  | GO:1901363 heterocyclic compound binding | - | - | - | - | - | - | - | - | 6.50E-03 | - |
| hsa-mir-143 | GO:0097159 organic cyclic compound binding | - | - | - | - | - | - | - | - | 6.50E-03 | - |
|  | GO:0043167 ion binding | - | - | - | - | - | - | - | - | 3.49E-02 | - |
|  | GO:0008134 transcription factor binding | - | - | - | 7.58E-06 | - | - | - | 5.70E-03 | - | - |
|  | GO:0000979 RNA polymerase II core promoter sequence-specific DNA binding | - | - | - | - | - | 2.03E-02 | - | 2.59E-02 | - | - |
| hsa-let-7c | GO:1990841 promoter-specific chromatin binding | - | 2.67E-02 | - | - | - | - | - | - | - | - |
|  | GO:0003682 chromatin binding | - | 2.67E-02 | - | - | - | - | - | - | - | - |
|  | GO:1901363 heterocyclic compound binding | - | 2.89E-02 | - | - | - | - | - | - | - | - |
|  | GO:0008134 transcription factor binding | 1.03E-02 | 4.60E-02 | - | - | - | - | 3.30E-02 | - | - | - |
|  | GO:0000900 translation repressor activity, mRNA regulatory element binding | - | 4.60E-02 | - | - | - | - | 3.30E-02 | 3.11E-02 | - | - |
| hsa-mir-107 | GO:0140096 catalytic activity, acting on a protein | 1.03E-02 | 4.60E-02 | - | - | - | - | - | - | - | - |
|  | GO:0016773 phosphotransferase activity, alcohol group as acceptor | 1.03E-02 | - | - | - | - | - | - | 3.11E-02 | - | - |
|  | GO:0005515 protein binding | 2.00E-03 | - | - | - | - | - | - | - | - | - |
|  | GO:0003676 nucleic acid binding | 1.64E-06 | - | - | - | - | - | 1.60E-02 | - | - | 8.00E-03 |
|  | GO:1901363 heterocyclic compound binding | 2.54E-06 | - | - | - | - | - | - | 2.30E-02 | - | - |
| hsa-mir-378 | GO:0097159 organic cyclic compound binding | 2.89E-06 | - | - | - | - | - | - | 2.30E-02 | - | - |
|  | GO:0003677 DNA binding | 9.80E-04 | - | - | - | - | - | - | - | - | 1.87E-02 |
|  | GO:0003723 RNA binding | 8.70E-03 | - | - | - | - | - | 1.60E-02 | - | - | - |
|  | GO:0043167 ion binding | - | 6.50E-03 | - | 2.37E-02 | - | - | 1.82E-02 | 4.32E-02 | - | - |
|  | GO:0005515 protein binding | - | - | - | 1.26E-02 | - | - | 1.82E-02 | 4.93E-02 | - | 1.11E-02 |
| hsa-mir-133a | GO:0032427 GBD domain binding | - | - | - | 1.79E-02 | - | - | 1.82E-02 | 4.32E-02 | - | 2.12E-02 |
|  | GO:0005516 calmodulin binding | - | 3.80E-03 | - | - | - | - | 1.82E-02 | 4.93E-02 | - | - |
|  | GO:0035091 phosphatidylinositol binding | - | 4.50E-03 | - | - | 3.34E-02 | - | 1.82E-02 | - | - | - |
|  | GO:1901363 heterocyclic compound binding | 5.70E-04 | - | - | 1.84E-02 | - | - | 1.10E-04 | - | - | 1.97E-02 |
|  | GO:0097159 organic cyclic compound binding | 5.70E-04 | - | - | 1.84E-02 | - | - | 1.30E-04 | - | - | 1.97E-02 |
| hsa-mir-1 | GO:0005524 ATP binding | 5.70E-04 | - | - | - | - | - | - | 1.20E-03 | - | - |
|  | GO:0005515 protein binding | - | 3.85E-02 | - | - | - | - | 5.95E-09 | - | - | - |
|  | GO:0005488 binding | - | - | - | 4.90E-04 | - | - | 8.49E-05 | - | - | - |
|  | GO:0005515 protein binding | 1.00E-04 | - | 1.28E-05 | - | 2.10E-03 | 7.60E-04 | 3.80E-03 | - | 1.27E-05 | 8.10E-03 |
|  | GO:0005488 binding | 2.16E-02 | - | 1.28E-05 | 5.93E-05 | 7.30E-03 | - | - | - | 1.10E-03 | - |
| hsa-mir-30c | GO:0019899 enzyme binding | 2.16E-02 | - | - | - | - | - | 3.90E-04 | - | 2.42E-05 | - |
|  | GO:0042802 identical protein binding | 5.10E-03 | - | 3.70E-03 | - | - | - | - | - | - | - |
|  | GO:0098772 molecular function regulator | - | - | - | - | 3.14E-02 | - | - | - | 3.80E-04 | - |
|  | GO:0005488 binding | 1.70E-04 | - | - | - | 1.42E-02 | - | 1.30E-03 | - | 1.70E-03 | 1.11E-02 |
|  | GO:0046332 SMAD binding | 5.70E-04 | - | - | - | - | - | 4.70E-04 | 7.70E-03 | - | - |
| hsa-mir-16 | GO:0005515 protein binding | 7.40E-04 | 3.70E-03 | - | - | - | - | 2.90E-04 | - | - | - |
|  | GO:0019838 growth factor binding | - | 3.70E-03 | - | - | - | - | - | 7.70E-03 | 4.74E-02 | - |
|  | GO:0008092 cytoskeletal protein binding | 5.70E-04 | - | 1.39E-02 | - | - | - | - | - | - | - |
|  | GO:0003676 nucleic acid binding | - | - | - | 9.80E-03 | - | - | - | 6.20E-04 | - | - |
|  | GO:0003723 RNA binding | - | - | - | 1.21E-02 | - | - | - | 9.09E-07 | - | - |
| hsa-mir-30a | GO:0019899 enzyme binding | - | - | - | 1.94E-02 | - | - | - | - | 4.60E-03 | - |
|  | GO:1901363 heterocyclic compound binding | - | - | - | 5.90E-03 | - | - | - | - | - | - |
|  | GO:0097159 organic cyclic compound binding | - | - | - | 5.90E-03 | - | - | - | - | - | - |
|  | GO:0051575 5’-deoxyribose-5-phosphate lyase activity | - | - | - | - | 2.80E-03 | - | - | - | - | - |
|  | GO:0008134 transcription factor binding | - | - | - | - | - | - | - | 2.84E-02 | - | - |
| hsa-let-7i | GO:0140297 DNA-binding transcription factor binding | - | - | - | - | - | - | - | 4.46E-02 | - | - |
|  | GO:0140110 transcription regulator activity | - | - | - | - | - | - | - | 4.46E-02 | - | - |
|  | GO:0042826 histone deacetylase binding | - | - | - | - | - | - | - | 4.46E-02 | - | - |
|  | GO:0035173 histone kinase activity | 2.30E-05 | 2.34E-05 | - | - | 2.36E-05 | - | 2.17E-05 | 4.17E-07 | - | 2.70E-05 |
|  | GO:0140097 catalytic activity, acting on DNA | 5.20E-04 | 7.50E-03 | - | - | - | - | - | 5.05E-05 | - | - |
| hsa-mir-24 | GO:1901363 heterocyclic compound binding | 1.40E-03 | 7.50E-03 | - | - | - | - | - | 2.80E-04 | - | - |
|  | GO:0005515 protein binding | - | - | 2.10E-02 | - | 5.30E-03 | 6.80E-04 | - | - | - | - |
|  | GO:0003684 damaged DNA binding | 2.90E-04 | - | - | - | - | - | - | 1.46E-05 | - | - |
|  | GO:1901363 heterocyclic compound binding | - | - | 3.00E-03 | - | - | - | 4.04E-02 | 1.03E-02 | - | - |
|  | GO:0140110 transcription regulator activity | - | - | 3.00E-03 | - | - | - | 4.04E-02 | 7.70E-03 | - | - |
| hsa-mir-95 | GO:0097159 organic cyclic compound binding | - | - | 3.00E-03 | - | - | - | 4.04E-02 | 1.03E-02 | - | - |
|  | GO:0003676 nucleic acid binding | - | - | 3.00E-03 | - | - | - | 3.42E-02 | 5.20E-03 | - | - |
|  | GO:0043565 sequence-specific DNA binding | - | - | 3.00E-03 | - | - | - | - | 1.03E-02 | - | - |

# REFERENCES

Altman, N. S. (1992). An introduction to kernel and nearest-neighbor nonparametric regression. *The American Statistician* 46, 175–185

Bennasar, M., Hicks, Y., and Setchi, R. (2015). Feature selection using joint mutual information maximisation. *Expert Systems with Applications* 42, 8520–8532

Breiman, L. (2005). Random Forests. *Machine Learning* 45, 5–32

Brown, G., Pocock, A., Zhao, M., and Lujan, M. (2012a). Conditional likelihood maximisation: a unifying framework for information theoretic feature selection. *The Journal of Machine Learning Research* 13, 27–66

Brown, G., Pocock, A., Zhao, M. J., and Lujan, M. (2012b). Conditional Likelihood Maximisation: A Unifying Framework for Information Theoretic Feature Selection. *Bioinformatics* 13, 27–66

Cortes, C. and Vapnik, V. (1995). Support-vector networks. *Machine Learning* 20, 273–297. doi:10.1007/ BF00994018

Fleuret, F. (2004). Fast binary feature selection with conditional mutual information. *Journal of Machine Learning Research* 5, 1531–1555. 00621

George, H. and Langley, J. P. (1995). Estimating Continuous Distributions in Bayesian Classifiers.

*Proceedings of the Eleventh Conference on Uncertainty in Artificial Intelligence* 69, 338–345

Hsu, C. W. and Lin, C. J. (2002). A comparison of methods for multiclass support vector machines. *IEEE Transactions on Neural Networks* 13, 415–425

Jakulin, A. (2005). Machine learning based on attribute interactions. *Univerza v Ljubljani*

Peng, H., Long, F., and Ding, C. (2005). Feature selection based on mutual information: criteria of max-dependency, max-relevance, and min-redundancy. *IEEE Transactions on Pattern Analysis and Machine Intelligence* 27, 1226–1238

Quinlan, J. R. (1986). Induction of decision trees. *Machine Learning* 1, 81–106

Quinlan, J. R. (1993). *C4.5: Programs for Machine Learning* (San Francisco, CA, USA: Morgan Kaufmann Publishers Inc.)

Troyanskaya, O. G., Garber, M. E., Brown, P. O., Botstein, D., and Altman, R. B. (2002). Nonparametric methods for identifying differentially expressed genes in microarray data. *Bioinformatics* 18, 1454–146.
